# Transcriptional Profiles of Mitochondrial Dynamics Markers Are Disturbed in Adrenal Glands of Stressed Adult Male Rats

**DOI:** 10.3390/life13071457

**Published:** 2023-06-27

**Authors:** Isidora M. Keselj, Filip N. Bozic, Miodrag M. Vucinic, Dusan Lalosevic, Tatjana S. Kostic, Silvana A. Andric

**Affiliations:** 1 Laboratory for Reproductive Endocrinology and Signaling, Laboratory for Chronobiology and Aging, CeRES, DBE, Faculty of Sciences, University of Novi Sad, 21000 Novi Sad, Serbia; isidora.keselj@dbe.uns.ac.rs (I.M.K.); flpbzc@gmail.com (F.N.B.); miodrag.vucinic94@gmail.com (M.M.V.); tatjana.kostic@dbe.uns.ac.rs (T.S.K.); 2 Medical Faculty, University of Novi Sad, 21000 Novi Sad, Serbia

**Keywords:** adrenal gland, mitochondrial dynamics markers, PGC1, PPARa, PPARd, MFN1/2, OPA1, FIS1, DRP1, stress

## Abstract

Mitochondrial dynamics plays a significant role in shaping the mitochondrial network and maintaining mitochondrial function. Imbalanced mitochondrial dynamics can cause mitochondrial dysfunction leading to a wide range of diseases/disorders. The aim of this study was to investigate the expression of mitochondrial dynamics markers and regulatory molecules in whole adrenal glands, cortices, and medullae obtained from adult male rats exposed to acute and repeated psychophysical stress, the most common stress in human society. The transcriptional profiles of most of the mitochondrial dynamics markers investigated here were altered: 81%-(17/21) in the whole adrenal gland, 76.2%-(16/21) in the adrenal cortex, and 85.7%-(18/21) in the adrenal medulla. Changes were evident in representatives of every process of mitochondrial dynamics. Markers of mitobiogenesis were changed up to 62.5%-(5/8) in the whole adrenal gland, 62.5%-(5/8) in the adrenal cortex, and 87.5%-(7/8) in the adrenal medulla. Markers of mitofusion were changed up to 100%-(3/3) in the whole adrenal gland, 66.7%-(5/8) in the adrenal cortex, and 87.5%-(7/8) in the adrenal medulla, while all markers of mitofission and mitophagy were changed in the adrenal glands. Moreover, almost all markers of mitochondrial functionality were changed: 83.3%-(5/6) in the whole adrenal, 83.3%-(5/6) in the cortex, 66.7%-(4/6) in the medulla. Accordingly, the study highlights the significant impact of acute and repeated stress on mitochondrial dynamics in the adrenal gland.

## 1. Introduction

The mitochondrial network, as an essential part of cell homeostasis, is regulated by the fascinating processes of mitochondrial dynamics, including mitochondrial biogenesis, mitofusion, mitofission, and mitophagy [1,2,3,4]. The mitochondrial network is also essential for the maintenance of cellular homeostasis and survival during stress by regulating cellular signaling, metabolism, and apoptosis. During stress, mitochondria undergo dynamic changes in their morphology, distribution, and function [5,6,7]. All these changes are regulated by a complex network of signaling pathways and involve a multistep molecular event for the renewal, adaptation, or expansion of the mitochondrial network [8,9,10]. Mitochondrial dynamics plays a critical role in the proper functioning of cells, including those in the adrenal gland. The main molecular markers of mitochondrial dynamics include the main markers of mitochondrial biogenesis (PPAR gamma coactivator α (PGC1α), PPAR gamma coactivator β (PGC1β), nuclear respiratory factor 1 (NRF1), nuclear respiratory factor 2 (NRF2), transcription factor A, mitochondrial (TFAM)), mitofusion (mitofusin 1 (MFN1), mitofusin 2 (MFN2)), mitochondrial dynamin-like GTPase (OPA1), mitofission (dynamin-related protein 1 (DRP1), fission, mitochondrial 1 (FIS1)), mitophagy (PTEN induced kinase 1 (PINK1), parkin RBR E3 ubiquitin protein ligase (PARKIN)) and markers of respiratory chain function. Furthermore, the intricate and complex signaling pathways [2,8,10] necessary to maintain homeostasis in the mitochondrial network are capable of transmitting a diverse range of environmental signals, such as stress [11,12], temperature [13], energy deprivation, and nutrient availability [10]. However, the dysregulation of mitochondrial dynamics has been implicated in a variety of stress-related disorders, including neurodegenerative diseases, cancer, and metabolic disorders [5,6].

Stress is a natural response of an organism that aids in survival and the maintenance of balance in the body [14]. However, if stress becomes chronic or repetitive, it can lead to several diseases [5,6,7,15,16]. The adrenal gland is a key player in the response to stress, and mitochondrial function is essential for its proper functioning. Adrenal cells have high energy demands due to the steroid hormone synthesis pathway being heavily reliant on ATP production through oxidative phosphorylation in mitochondria. Stress-induced changes in the adrenal mitochondria are mediated by a complex interplay of signaling pathways, involving hormonal and neural signals, as well as nutrient availability and cellular energy status [17].

Imbalanced mitochondrial dynamics can cause mitochondrial dysfunction, leading to a wide range of diseases/disorders. Since the adrenal glands are essential for maintaining the body’s stress response and metabolism [5,6], the aim of this study was to investigate the transcriptional profile of mitochondrial dynamics markers and regulatory molecules in whole adrenal glands as well as in cortices and medullae obtained from adult male rats exposed to acute as well as repeated psychophysical stress, the most common stress in human society.

## 2. Materials and Methods

All samples, commercial reagents, primers, antibodies, and software that were used in this study are given in Supplementary Table S1.

All experiments were performed in the Laboratory for Reproductive Endocrinology and Signaling and Laboratory for Chronobiology and Aging, Faculty of Sciences at the University of Novi Sad (https://wwwold.dbe.pmf.uns.ac.rs/en/nauka-eng/lares; accessed on 5 June 2023). All the methods used in this study have been previously reported by our group (for all references please see [18,19,20] and follow the relevant guidelines and regulations).

### 2.1. Statement of Institutional Review Board

The Committee of the Faculty of Sciences, University of Novi Sad, Novi Sad, Serbia approved this manuscript.

### 2.2. A Statement That the Authors Complied with ARRIVE Guidelines and Institutional Animal Care and Use Committee Guidelines

The authors complied with ARRIVE guidelines, and all experiments were in adherence to the ARRIVE guidelines. Furthermore, all experimental protocols were approved (statement no. 04-81/114, dated 25 September 2020) by the local Ethical Committee on Animal Care and Use of the University of Novi Sad operating under the rules of the National Council for Animal Welfare and the National Law for Animal Welfare (copyright March 2009), following the NRC publication “Guide for the Care and Use of Laboratory Animals” and NIH’s “Guide for the Care and Use of Laboratory Animals”.

### 2.3. Animals and Experimental Model of Stress

**Figure 1:**
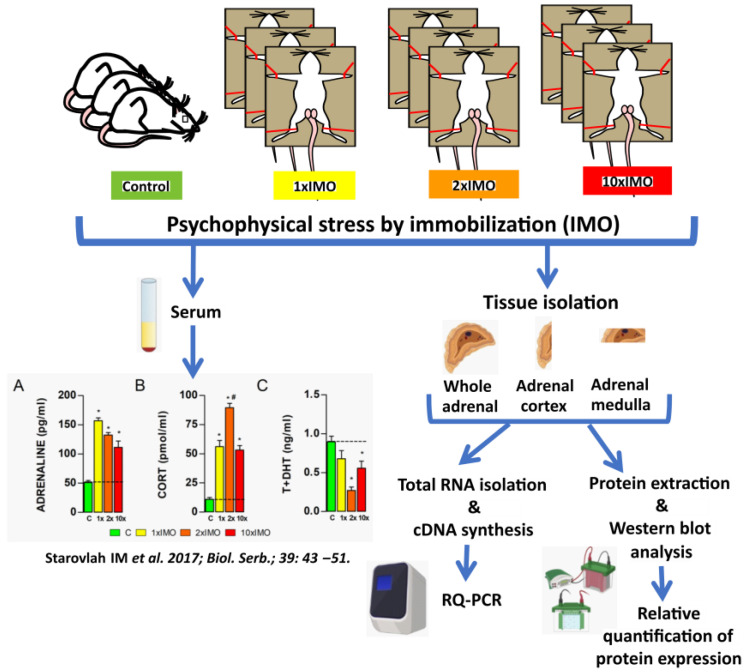
Experimental design of psychophysical stress by immobilization used to assess profiles of mitochondrial dynamics markers expression in the whole adrenal gland, the adrenal cortex, and adrenal medulla. Previously published results [19] showed that IMO stress induces an increase of circulating adrenaline (**A**) and corticosterone (**B**) levels, and in the same time a decrease of androgens (T+DHT) levels (**C**). Statistical significance was set at level of *p* < 0.05: * vs. control group and # vs. 1 × IMO group.

Adult, three-month-old male Wistar rats were used in all the experiments. All the animals were bred and raised in the accredited Animal Facility of the Faculty of Sciences, University of Novi Sad, Serbia. The Animal Facility provided controlled environmental conditions (22 ± 2 °C; 14 h light and 10 h dark cycle; lights on at 07:00 AM) with food and water available ad libitum. The experimental model of psychophysical stress by immobilization (IMO) was performed using the method of Kvetnansky [21] with some modifications previously described by our group [18,19] (Figure 1). Briefly, stressed (IMO) rats were bound in a supine position to a wooden board by fixing the rats’ limbs using thread, while the head motion was not limited. In each experiment, unstressed, freely moving animals were used as a control group. To analyze the effect of psychophysical stress, animals were subjected to immobilization stress once (1 × IMO) for two hours, or repeatedly for two (2 × IMO) and ten (10 × IMO) consecutive days, in the duration of two hours at the same period during the day (from 07:00 AM to 9:00 AM). At the end of the experimental period, control and stressed animals were quickly decapitated without anesthesia, and adrenal glands were isolated. In each experiment, the control and experimental group consisted of four animals. The experiments were repeated twice.

### 2.4. Isolation of Adrenal Glands

At the end of the experimental period, control and stressed animals were quickly decapitated without anesthesia, and adrenal glands were isolated. After the isolation, whole adrenal glands were immediately frozen, or the whole adrenal glands were used for the separation of the adrenal cortices and adrenal medullae. After the isolation of the whole adrenal glands, they were cut in the middle using a sharp razor blade. Using the magnifying glass, anatomic structures of the adrenal cortex and medulla were distinguished. Using sharp pointed tweezers, a pinch of the adrenal medulla and adrenal cortex were isolated and immediately frozen on dry ice.

### 2.5. Isolation of RNA and cDNA Synthesis

Whole adrenal gland, adrenal cortex, and adrenal medulla tissue samples were stored at −70 °C until they were used for the isolation of total RNA. Total RNA isolation was performed using the TRIzol™ Reagent following the protocol recommended by the manufacturer (Invitrogen™, Thermo Fisher Scientific Inc., Waltham MA, USA, www.thermofisher.com; accessed on 5 June 2023). To eliminate DNA from the samples, DNase I (RNase-free) treatments were performed according to the manufacturer’s instructions (New England Biolabs, Ipswich, MA, USA, www.neb.com; accessed on 5 June 2023). The concentration and purity of isolated total RNA were measured using the BioSpec nano-spectrophotometer (Shimadzu, Kyoto, Japan, www.shimadzu.com; accessed on 5 June 2023). Furthermore, the first-strand cDNA was synthesized using the High-Capacity Kit for cDNA Preparation following the manufacturer’s protocol (Thermo Fisher Scientific, Waltham, MA, USA, www.thermofisher.com; accessed on 5 June 2023). The quality of RNA and DNA integrity was checked using control primers for *Gapdh*, as described previously by our group (for reference, please see [18,19,22]).

### 2.6. Relative Quantification of Gene Expression

Real-time PCR was used for the relative expression of the genes and was quantified using SYBR^®^Green-based chemistry from Applied Biosystems (Thermo Fisher Scientific, Waltham, MA, USA, www.thermofisher.com; accessed on 5 June 2023). For each reaction, 15–20 ng of cDNA was used (calculated from starting RNA) in the volume of 2.5 μL with specific primers at a final concentration of 500 nM. Primer sequences used for real-time PCR analysis and Ct values, as well as GenBank accession codes for full gene sequences (www.ncbi.nlm.nih.gov/sites/entrez; accessed on 5 June 2023), are given in Supplementary Tables S2–S7. Relative gene expression quantification of *Gapdh* was measured in each sample and used to correct the variations in cDNA content between samples. Relative quantification of each gene was performed in duplicate, two times for each independent, in vivo experiment. The real-time PCR reactions were carried out in the Eppendorf Mastercycler ep realplex 4 and post-run analyses were performed using Mastercycler^®^ ep realplex software version 1.0 (for reference, please see [18,19,22]).

### 2.7. Relative Quantification of Protein Expression

Whole adrenal glands were frozen and stored at −80 °C until protein extraction. Tissue was lysed, and Western blot analysis was performed, as described previously in [18,19,20]. Immune-reactive bands were detected using MyECL Imager (Thermo Fisher Scientific Inc.) and analyzed as two-dimensional images using Image J version 1.48 (http://rsbweb.nih.gov/ij/download.html; accessed on 5 June 2023). The optical density of images was expressed as volume adjusted for the background, which provided arbitrary units of adjusted volume. The normalization of the data was carried out using ACTIN protein expression as the endogenous control. Immune detection was performed with different antibodies (all details are listed in Table S8).

### 2.8. Statistical Analysis

Results of the experiments are represented by group means ± SEM values of the individual variation from two independent experiments with four animals per group. Results from each experiment were analyzed by one-way ANOVA for group comparison, followed by Student–Newman–Keuls multiple range test. All the statistical analyses were performed using GraphPad Prism 8 Software (GraphPad Software 287 Inc., La Jolla, CA, USA). In all cases, a *p*-value < 0.05 was considered to be statistically significant.

## 3. Results

In order to understand the connection between stress and mitochondrial dynamics markers in the adrenal gland, an immobilization stress (IMO) period of 2 h was applied to the adult male rats once (1 × IMO) as an acute stress, or for two (2 × IMO) and ten (10 × IMO) consecutive days as a repeated stress [18,19]. After the immobilization period, the transcriptional profiles of mitochondrial dynamic markers were obtained in the whole adrenal gland tissue, as well as the adrenal cortex and adrenal medulla tissues. In addition, the expression of the proteins involved in the maintenance and regulation of mitochondrial dynamics were studied.

### 3.1. The Transcriptional Profiles of Mitochondrial Biogenesis Markers Were Changed in the Adrenal Gland of Stressed Rats

**Figure 2:**
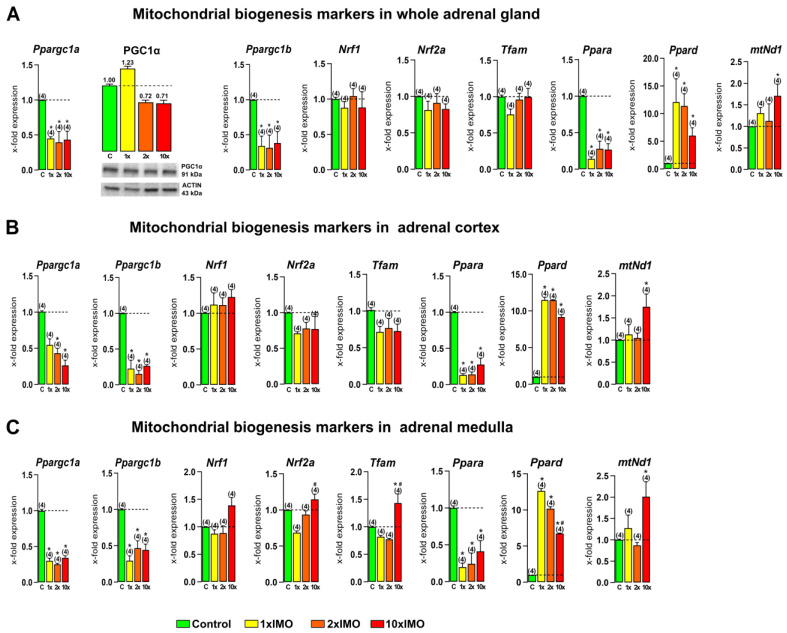
Transcriptional profiles of mitochondrial biogenesis markers in the whole adrenal gland, adrenal cortex, and medulla tissues after one, two, and ten times repeated psychophysical stress of the entire organism. Whole adrenal gland tissue (**A**) as well as the adrenal cortex (**B**) and medulla (**C**) tissues were isolated from undisturbed rats, as well as acutely (1 × IMO), and repeatedly (2 × IMO and 10 × IMO) stressed rats. Tissue was further used for RNA isolation followed by the analysis of the transcriptional profile of mitochondrial biogenesis markers. Whole adrenal gland tissue was used for PGC1 protein expression analysis. The representative blots are shown as panels. Data from scanning densitometry were normalized to ACTIN (endogenous control). Values are shown as bars above the photos of blots. The data bars represent mean ± SEM values of two independent in vivo experiments (number in brackets above the bars represents number of analyzed animals). Statistical significance was set at level *p* < 0.05: * vs. control group and # vs. 1 × IMO group.

Our analysis of the relative expression of the main mitochondrial dynamic markers showed a disturbed expression in the whole adrenal gland (five out of eight or 62.5%), as well as in the adrenal cortex (five out of eight or 62.5%), while the most prominent change was detected in the adrenal medulla (seven out of eight or 87.5%) (Figure 2). The level of *Ppargc1a*, encoding PGC1, the master regulator of mitochondrial biogenesis [1,2], was significantly decreased in all of the stressed groups in the whole adrenal gland (1 × IMO 2.2-fold, 2 × IMO 2.8-fold, 10 × IMO 2.3-fold), as well as in the adrenal cortex (1 × IMO 1.8-fold, 2 × IMO 2.3-fold, 10 × IMO 3.8-fold) and the adrenal medulla (1 × IMO 3.3-fold, 2 × IMO 4.0-fold, 10 × IMO 2.9-fold). On the other hand, the protein expression of the same marker was not statistically different between the stressed and control groups. Similarly, the decreased transcription of *Ppargc1b* was detected in the whole adrenal gland of all the analyzed stressed groups (1 × IMO 2.9-fold, 2 × IMO 3.2-fold, 10 × IMO 2.6-fold), as well as in the adrenal cortex (1 × IMO 4.4-fold, 2 × IMO 6.6-fold, 10 × IMO 3.8-fold) and adrenal medulla (1 × IMO 3.4-fold, 2 × IMO 2.1-fold, 10 × IMO 2.3-fold). The transcriptional profiles of the mitochondrial dynamic markers *Nrf1* and *Nrf2a* were not changed in the whole adrenal gland, adrenal cortex, and adrenal medulla of the stressed rats compared to the control group, while only the 10 × IMO stress treatment increased *Nrf2a* transcription in the adrenal medulla compared to the 1 × IMO group (1.7-fold). Furthermore, the transcriptional profile of the mitochondrial transcription factor *Tfam* was increased only in the adrenal medulla of the 10 × IMO group compared to that of both the control (1.4-fold) and the 1 × IMO (1.7-fold) group. The decreased transcription of the master regulator of mitochondrial biogenesis was followed by a decreased transcription of its downstream target *Ppara* in the whole adrenal glands of the acutely (7.1-fold) and repeatedly stressed rats (2 × IMO 3.6-fold, 10 × IMO 3.7-fold). This decrease in the *Ppara* transcript was also detected in the adrenal cortex (1 × IMO 7.5-fold, 2 × IMO 7.1-fold, 10 × IMO 3.6-fold) and adrenal medulla (1 × IMO 5.1-fold, 2 × IMO 4.1-fold, 10 × IMO 2.4-fold). On the other hand, an increased transcriptional profile of *Ppard* was detected in the whole adrenal gland of all the stressed groups (1 × IMO 12.1-fold, 2 × IMO 11.4-fold, 10 × IMO 6.0-fold), as well as in the adrenal cortex (1 × IMO 11.5-fold, 2 × IMO 11.5-fold, 10 × IMO 9.2-fold) and in the adrenal medulla (1 × IMO 12.7-fold, 2 × IMO 10.2-fold, 10 × IMO 6.7-fold). Furthermore, only the 10 × IMO stress treatment increased the transcription of *mtNd1* in the whole adrenal gland (1.7-fold), adrenal cortex (1.8-fold), and adrenal medulla (2.0-fold).

### 3.2. Repeated Psychophysical Stress Increased the Transcriptional Profile of Mitochondrial Fusion and Architecture Markers in the Adrenal Gland

**Figure 3:**
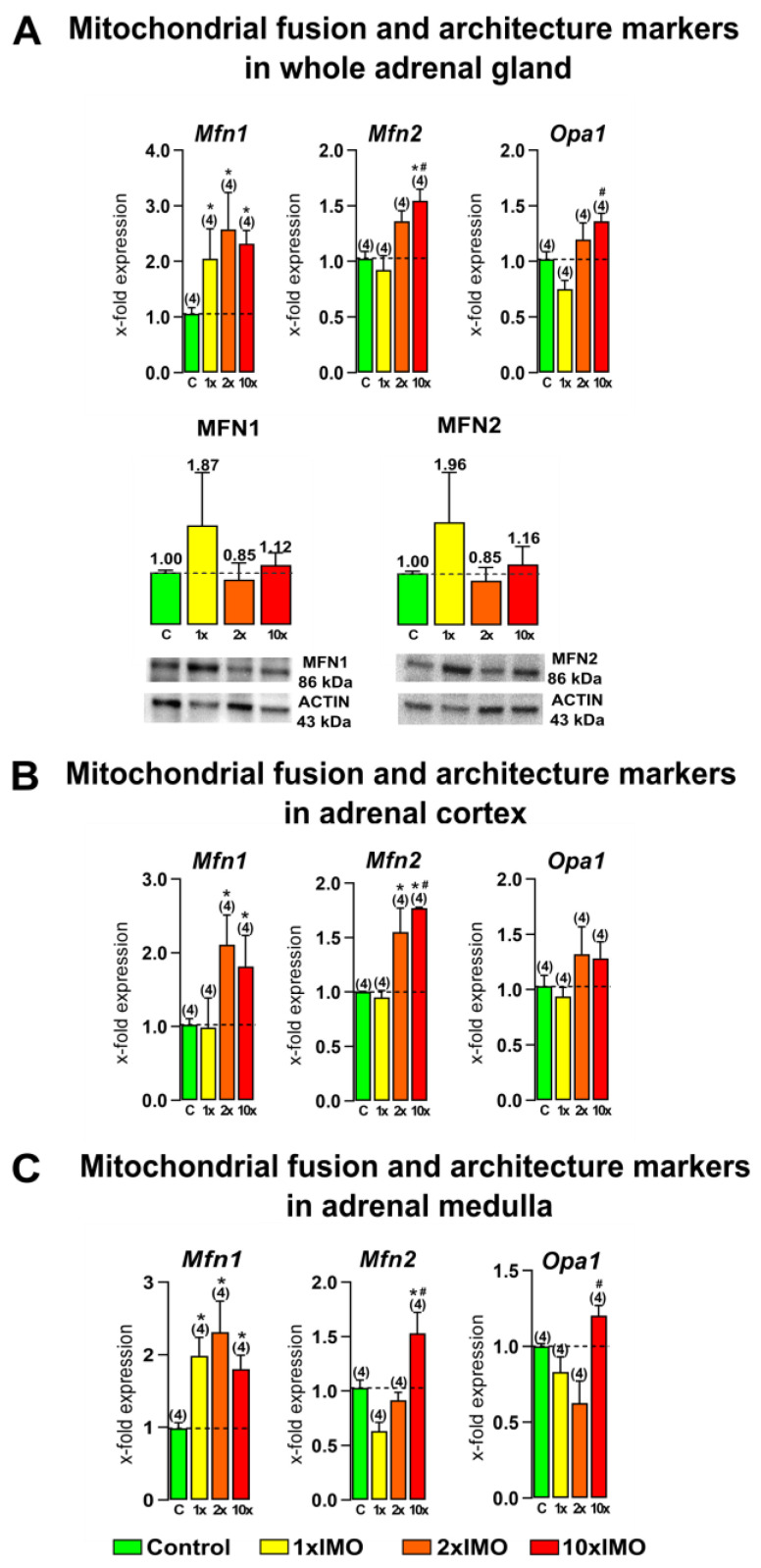
Ten repeated psychophysical stress incidents increased the expression of mitochondrial fusion and architecture markers in the whole adrenal gland, adrenal cortex, and medulla tissues. Whole adrenal gland tissue (**A**), as well as the adrenal cortex (**B**) and medulla (**C**) tissues, were isolated from undisturbed rats, as well as acutely (1 × IMO) and repeatedly (2 × IMO and 10 × IMO) stressed rats. Tissue was further used for RNA isolation followed by the analysis of the transcriptional profile of mitochondrial fusion markers. Whole adrenal gland tissue was used for MFN1 and MFN2 protein expression analyses. The representative blots are shown as panels. Data from scanning densitometry were normalized to ACTIN (endogenous control). Values are shown as bars above the photos of blots. The data bars represent mean ± SEM values of two independent in vivo experiments (number in brackets above the bars represents number of analyzed animals). Statistical significance was set at level of *p* < 0.05: * vs. control group and # vs. 1 × IMO group.

The levels of transcripts for all of the mitochondrial fusion and architecture markers (three out of three or 100%) in the adrenal gland were changed after repeated psychophysical stress by immobilization (Figure 3). Our results showed that all of the types of stress significantly increased *Mfn1* transcription in the whole adrenal gland (1 × IMO 2.0-fold, 2 × IMO 2.6-fold, 10 × IMO 2.3-fold) and the adrenal medulla (1 × IMO 2.0-fold, 2 × IMO 2.3-fold, 10 × IMO 1.8-fold), while the transcription of the same marker was increased in the adrenal cortex only after repeated stress (2 × IMO 2.1-fold, 10 × IMO 1.8-fold). The increased transcriptional profile of *Mfn2* was detected after the 10 × IMO stress treatment (10 × IMO) in the whole adrenal gland (1.5-fold vs. control and 1.7-fold vs. 1 × IMO) as well as in the adrenal cortex (1.8-fold vs. control and 1.9-fold vs. 1 × IMO) and medulla (1.5-fold vs. control and 2.4-fold vs. 1 × IMO). Moreover, an increased level of the *Mfn2* transcript was detected after 2 × IMO stress treatment (2 × IMO) only in the adrenal cortex tissue (1.6-fold). On the other hand, the protein expression of both markers of mitochondrial fusion and architecture were not changed in the whole adrenal glands of the acutely (1 × IMO) and repeatedly (2 × IMO and 10 × IMO) stressed rats. Furthermore, an increased transcriptional profile of the *Opa1* transcript was detected after the 10 × IMO treatment in the whole adrenal gland (1.8-fold) and adrenal medulla (1.5-fold) compared to the 1 × IMO treatment.

### 3.3. The Transcriptional Profiles of Mitochondrial Fission Markers Were Increased in the Adrenal Gland after Ten Repeated Stress Incidents

**Figure 4:**
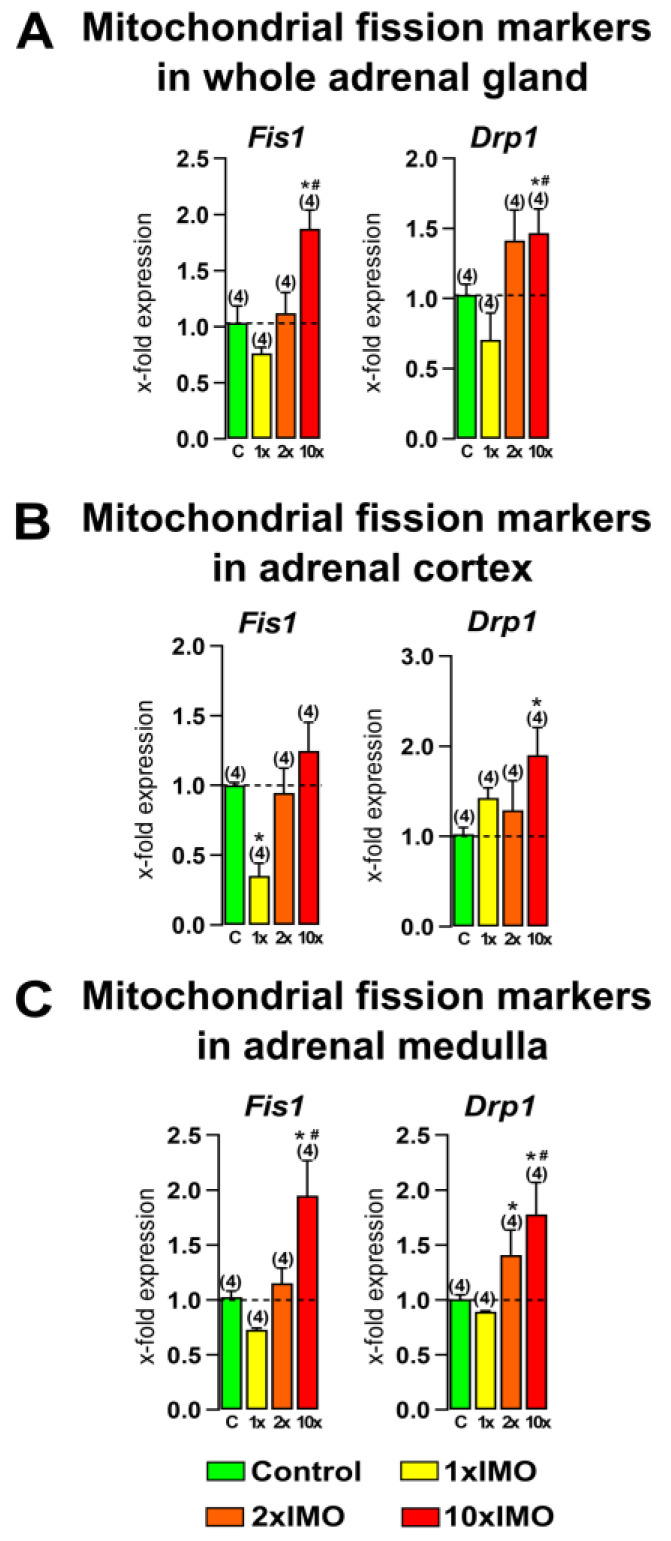
Different transcriptional profiles of mitochondrial fission markers in the whole adrenal gland, adrenal cortex, and medulla tissues after one, two, and ten times repeated psychophysical stress of the entire organism. Whole adrenal gland tissue (**A**), as well as the adrenal cortex (**B**) and medulla (**C**) tissues, were isolated from undisturbed rats, as well as acutely (1 × IMO) and repeatedly (2 × IMO and 10 × IMO) stressed rats. Tissue was further used for RNA isolation followed by an analysis of the transcriptional profile of mitochondrial fission markers. The data bars represent mean ± SEM values of two independent, in vivo experiments (number in brackets above the bars represents number of analyzed animals). Statistical significance was set at level of *p* < 0.05: * vs. control group and # vs. 1 × IMO group.

The levels of transcript for both the analyzed mitochondrial fission markers (two out of two or 100%) in the adrenal gland were changed after repeated psychophysical stress by immobilization (Figure 4). The relative expression of *Fis1* increased after the 10 × IMO treatment in the whole adrenal gland (1.9-fold vs. control and 2.5-fold vs. 1 × IMO) and adrenal medulla (2.0-fold vs. control and 2.7-fold vs. 1 × IMO). On the other hand, only the acute stress (1 × IMO) decreased the expression of *Fis1* in the adrenal cortex tissue (2.8-fold). The results show that the 10 × IMO stress treatment increased the transcription of *Drp1* in the whole adrenal gland (1.5-fold vs. control and 2.1-fold vs. 1 × IMO) and adrenal cortex (1.9-fold), while in the adrenal medulla, both types of repeated stress, the 2 × IMO treatment (1.4-fold) and the 10 × IMO treatment (1.8-fold vs. control and 2.0-fold vs. 1 × IMO) increased the transcription of *Drp1*.

### 3.4. Ten Repeated Psychophysical Stress Incidents Increased the Transcriptional Profile of Mitochondrial Autophagy Markers in the Adrenal Gland

**Figure 5:**
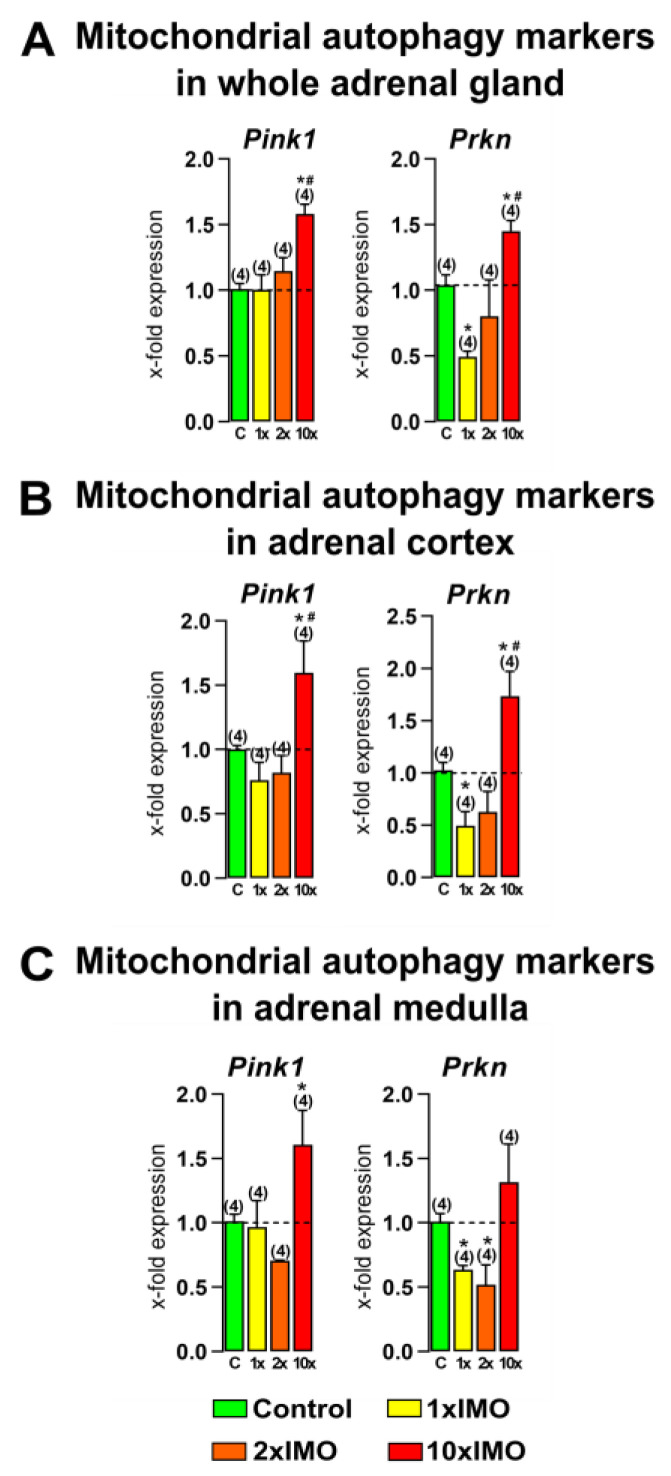
Acute and repeated psychophysical stress of the entire organism changed transcriptional profiles of mitochondrial autophagy markers in the whole adrenal gland, adrenal cortex, and medulla tissues. Whole adrenal gland (**A**) tissue, as well as adrenal cortex (**B**) and medulla (**C**) tissues, were isolated from undisturbed rats, as well as acutely (1 × IMO) and repeatedly (2 × IMO and 10 × IMO) stressed rats. Tissue was further used for RNA isolation followed by an analysis of the transcriptional profile of mitochondrial autophagy markers. The data bars represent mean ± SEM values of two independent, in vivo experiments (number in brackets above the bars represents number of analyzed animals). Statistical significance was set at level of *p* < 0.05: * vs. control group and # vs. 1 × IMO group.

The transcriptional analysis of the main mitochondrial autophagy markers showed that repeated stress increased the transcription of all of the analyzed markers (two out of two or 100%) in the adrenal gland tissue (Figure 5). The treatment with ten times repeated stress (10 × IMO) increased the transcription of *Pink1* in the whole adrenal gland (1.6-fold vs. control and 1.6-fold vs. 1 × IMO), adrenal cortex (1.6-fold vs. control and 2.1-fold vs. 1 × IMO), and adrenal medulla (1.6-fold). Moreover, the 10 × IMO treatment increased the transcription of *Prkn* in the whole adrenal gland (1.5-fold vs. control and 2.9-fold vs. 1 × IMO) and adrenal cortex (1.7-fold vs. control and 3.5-fold vs. 1 × IMO). On the other hand, the 2 × IMO stress treatment decreased the transcription of *Prkn* in the adrenal medulla tissue (1.9-fold), while acute stress decreased the transcription of the same transcript in the whole adrenal gland (2.0-fold), adrenal cortex (2.0-fold), and adrenal medulla (1.6-fold).

### 3.5. The Transcription of Mitochondrial Functionality Markers Was Changed in the Adrenal Gland of Stressed Rats

**Figure 6:**
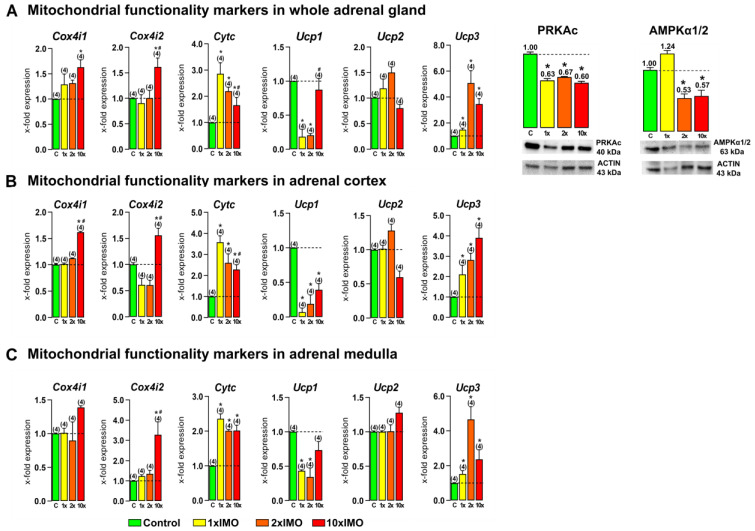
The transcriptional profiles of mitochondrial functionality markers are different after acute and repeated psychophysical stress of the entire organism in the whole adrenal gland, adrenal cortex, and medulla tissues. Whole adrenal gland (**A**) tissue as well as adrenal cortex (**B**) and medulla (**C**) tissues were isolated from undisturbed rats, as well as acutely (1 × IMO) and repeatedly (2 × IMO and 10 × IMO) stressed rats. Tissue was further used for RNA isolation followed by an analysis of the transcriptional profile of the main markers of mitochondrial functionality. Whole adrenal gland tissue was used for PRKAc and AMPKα1/2 protein expression analysis. The representative blots are shown as panels. Data from scanning densitometry were normalized to ACTIN (endogenous control). Values are shown as bars above the photos of blots. The data bars represent mean ± SEM values of two independent, in vivo experiments (number in brackets above the bars represents number of analyzed animals). Statistical significance was set at level of *p* < 0.05: * vs. control group and # vs. 1 × IMO group.

The levels of transcripts for five out of six (83.3%) of the mitochondrial functionality markers were changed in the adrenal gland after psychophysical stress by immobilization (Figure 6). The repeated stress (10 × IMO) treatment increased the transcription of *Cox4i1* in the whole adrenal gland (1.6-fold) and adrenal cortex (1.6-fold vs. control and 1 × IMO). Moreover, the 10 × IMO treatment increased the transcription of another analyzed subunit of COX4, *Cox4i2*, in the whole adrenal gland (1.6-fold vs. control and 1.8-fold vs. 1 × IMO), adrenal cortex (1.6-fold vs. control and 2.5-fold vs. 1 × IMO), and adrenal medulla (3.3-fold vs. control and 2.6-fold vs. 1 × IMO). On the other hand, all types of stress increased the transcription of *Cytc* in the whole adrenal gland (1 × IMO 2.9-fold, 2 × IMO 2.2-fold, 10 × IMO 1.7-fold and decreased 1.7-fold vs. 1 × IMO), adrenal cortex (1 × IMO 3.6-fold, 2 × IMO 2.6-fold, 10 × IMO 2.3-fold and decreased 1.6-fold vs. 1 × IMO), and adrenal medulla (1 × IMO 2.4-fold, 2 × IMO 2.0-fold, 10 × IMO 2.0-fold). Furthermore, psychophysical stress by immobilization changed the expression of *Ucp1* in the analyzed adrenal gland tissues. The acute (1 × IMO) and 2 × IMO treatments decreased the transcription of *Ucp1* in the whole adrenal gland (1 × IMO 5.4-fold, 2 × IMO 4.9-fold), adrenal cortex (1 × IMO 14.2-fold, 2 × IMO 5.3-fold), and adrenal medulla (1 × IMO 2.3-fold, 2 × IMO 2.9-fold). Additionally, the 10 × IMO treatment decreased the transcription of *Ucp1* in the adrenal cortex (2.6-fold) compared with the control group, while the 10 × IMO treatment increased the transcription of *Ucp1* in the whole adrenal gland (4.7-fold) compared to the 1 × IMO group. In contrast, both the acute (1 × IMO) and repeated (2 × IMO and 10 × IMO) stress treatments significantly increased the transcription of *Ucp3* in the whole adrenal gland (1 × IMO 1.5-fold, 2 × IMO 5.1-fold, 10 × IMO 3.5-fold), adrenal cortex (1 × IMO 2.1-fold, 2 × IMO 2.8-fold, 10 × IMO 3.9-fold), and adrenal medulla (1 × IMO 1.5-fold, 2 × IMO 4.7-fold, 10 × IMO 2.4-fold).

Furthermore, psychophysical stress by immobilization changed the expression of *Ucp1* in the analyzed adrenal gland tissues. The acute (1 × IMO) and 2 × IMO treatments decreased the transcription of *Ucp1* in the whole adrenal gland (1 × IMO 5.4-fold, 2 × IMO 4.9-fold), adrenal cortex (1 × IMO 14.2-fold, 2 × IMO 5.3-fold), and adrenal medulla (1 × IMO 2.3-fold, 2 × IMO 2.9-fold). Additionally, the 10 × IMO treatment decreased the transcription of *Ucp1* in the adrenal cortex (2.6-fold) compared with that of the control group, while the 10 × IMO treatment increased the transcription of *Ucp1* in the whole adrenal gland (4.7-fold) compared to that of the 1 × IMO group. In contrast, both the acute (1 × IMO) and repeated (2 × IMO and 10 × IMO) stress treatments significantly increased the transcription of *Ucp3* in the whole adrenal gland (1 × IMO 1.5-fold, 2 × IMO 5.1-fold, 10 × IMO 3.5-fold), adrenal cortex (1 × IMO 2.1-fold, 2 × IMO 2.8-fold, 10 × IMO 3.9-fold), and adrenal medulla (1 × IMO 1.5-fold, 2 × IMO 4.7-fold, 10 × IMO 2.4-fold) (Figure 5). Furthermore, an analysis of the protein expression of kinases important for mitochondrial functionality, PRKAc (Protein kinase C, catalytic subunit) and AMPKα1/2 (5′-AMP-activated protein kinase, α subunit), showed that both types of repeated stress (the 2 × IMO and 10 × IMO treatments) decreased the protein expression of mentioned kinases, but only the 1 × IMO stress treatment decreased the expression of PRKAc in the whole adrenal gland.

### 3.6. The Transcription of Adrenergic Receptors, Adrenergic Receptor Kinases, and Glucocorticoid Receptor Was Changed in the Adrenal Gland of Stressed Rats

**Figure 7:**
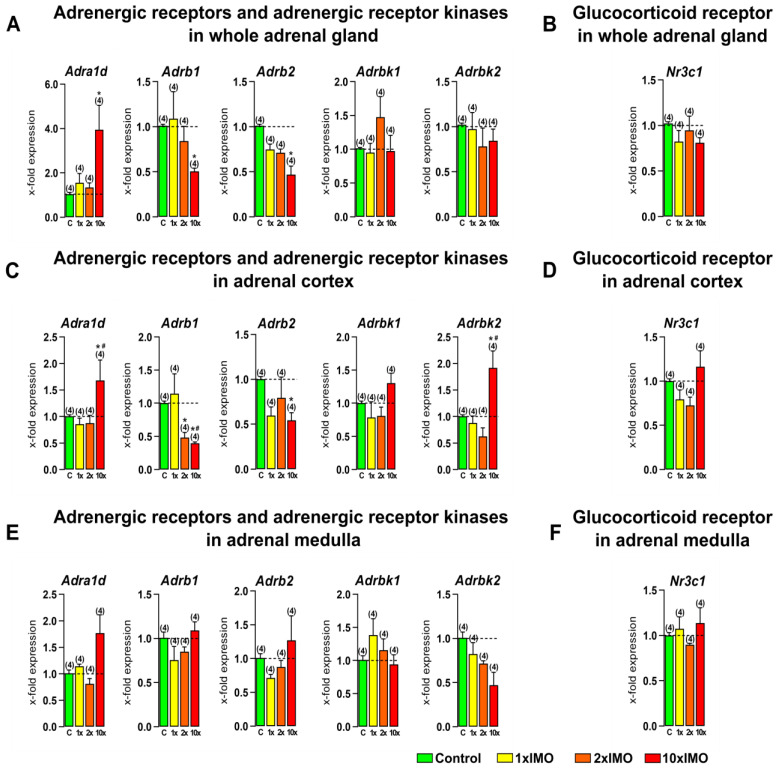
Acute and repeated psychophysical stress of the entire organism changed transcriptional profiles of adrenergic receptors and adrenergic receptor kinases but not glucocorticoid receptor in the whole adrenal gland, adrenal cortex, and medulla tissues. Whole adrenal gland (**A**,**B**) tissue as well as adrenal cortex (**C**,**D**) and medulla (**E**,**F**) tissues were isolated from undisturbed rats, as well as acutely (1 × IMO) and repeatedly (2 × IMO and 10 × IMO) stressed rats. Tissue was further used for RNA isolation followed by an analysis of the transcriptional profile of adrenergic receptors, adrenergic receptor kinases, and glucocorticoid receptor. The data bars represent mean ± SEM values of two independent, in vivo experiments (number in brackets above the bars represents number of analyzed animals). Statistical significance was set at level of *p* < 0.05: * vs. control group and # vs. 1 × IMO group.

The levels of transcripts for three out of five (60.0%) of the adrenergic receptors and adrenergic receptor kinases were changed in the adrenal gland after psychophysical stress by immobilization (Figure 7). The repeated stress 10 × IMO treatment increased the transcription of *Adra1d* in the whole adrenal gland (4.0-fold) and adrenal cortex tissue (1.7-fold vs. control and 2.0-fold vs. 1 × IMO). In contrast, the 10 × IMO stress treatment decreased the transcription of *Adrb1* in the whole adrenal gland (2.0-fold) and adrenal cortex (2.5-fold vs. control and 2.9-fold vs. 1 × IMO), while the 2 × IMO stress treatment decreased the transcription of the same marker only in the adrenal cortex tissue (2.1-fold). Moreover, the transcription of *Adrb2* was decreased after the 10 × IMO stress treatment in the whole adrenal gland (2.1-fold) and adrenal cortex tissue (1.9-fold). Furthermore, the same type of repeated stress (10 × IMO) increased the transcription of *Adrbk2* (1.9-fold vs. control and 2.2-fold vs. 1 × IMO) in the adrenal cortex. The transcriptional profile of the glucocorticoid receptor (*Nr3c1*) was not changed in any analyzed tissue after the acute (1 × IMO) and repeated (2 × IMO and 10 × IMO) stress treatments. 

**Figure 8:**
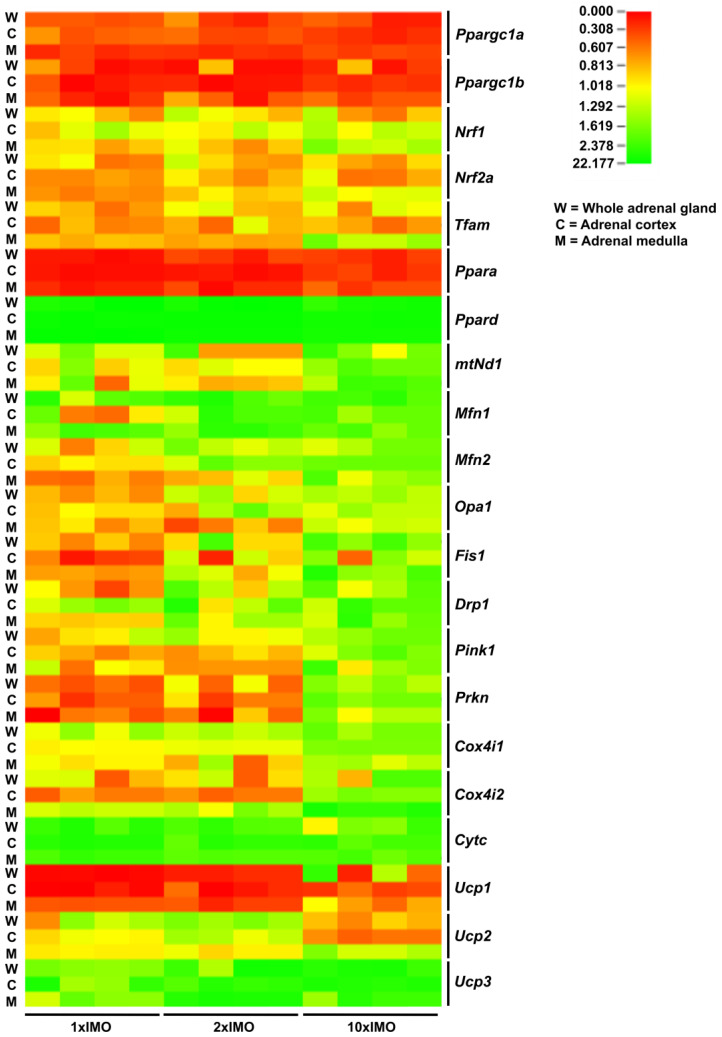
Heat map analysis of the transcriptional profile of the mitochondrial dynamics and functionality markers in whole adrenal gland (W) tissue as well as the adrenal cortex (C) and medulla (M) tissues isolated from undisturbed rats, as well as acutely (1 × IMO) and repeatedly (2 × IMO and 10 × IMO) stressed rats. The color gradient from red to green represents low to high expression levels, respectively.

A heat map analysis of the transcriptional profile of the mitochondrial dynamics and functionality markers clearly showed a distinction in the transcriptional profiles between some of the members of the same family (*Ppara* vs. *Ppard*) in the adrenal glands tissues obtained from acutely (1 × IMO) and repeatedly (2 × IMO and 10 × IMO) stressed rats (Figure 8).

## 4. Discussion

The mitochondrial network is formed by a complex interconnection of mitochondria within cells that plays a crucial role in maintaining mitochondrial function [1,2,3,4]. These processes of mitochondrial dynamics help to maintain mitochondrial quality control by redistributing damaged components among healthy mitochondria or by isolating damaged mitochondria for degradation. In healthy cells, the mitochondrial network is dynamic and adaptable, allowing cells to respond to changes in energy demands [5,6]. Besides many other important functions (please see the introduction), mitochondria play a crucial role in the body’s response to stress by increasing their activity to provide the extra energy that the body needs to cope with stress. On the other hand, excessive or prolonged stress can lead to damage to mitochondria and can affect their ability to produce energy, leading to mitochondrial dysfunction. This can contribute to a wide range of health problems, including chronic fatigue, metabolic disorders, and neurodegenerative diseases [5,6]. However, there is evidence suggesting that improving mitochondrial function can help to reduce the negative effects of stress on the body. Recent research has suggested that targeting the mitochondrial network may be a promising approach for treating mitochondrial dysfunction [23].

Giving the importance of the adrenal glands for maintaining the body’s stress response and metabolism [5,6], the aim of this study was to investigate the expression of mitochondrial dynamics markers and regulatory molecules in whole adrenal glands as well as in cortices and medullae obtained from adult male rats exposed to acute as well as repeated psychophysical stress, the most common stress in human society.

**Figure 9:**
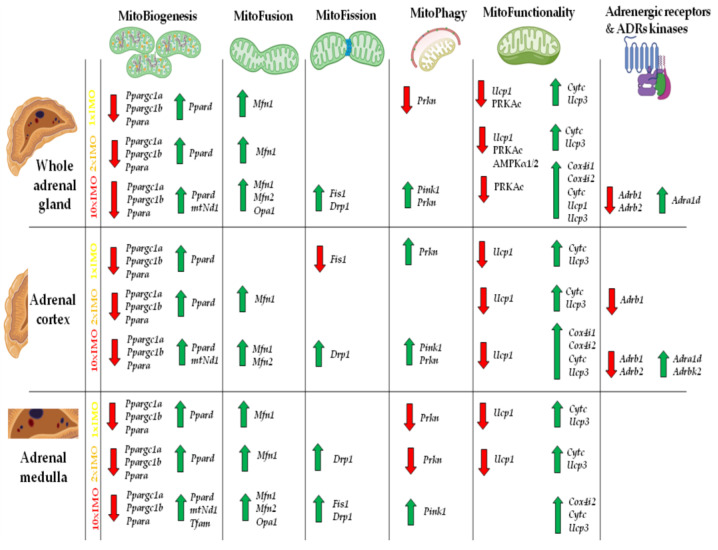
The transcriptional profile of mitochondrial dynamics and functionality markers, adrenergic receptors, and adrenergic receptor kinases in whole adrenal gland tissue as well as adrenal cortex and medulla isolated from undisturbed rats, as well as acutely (1 × IMO) and repeatedly (2 × IMO and 10 × IMO) stressed rats. The green up arrow symbol represents increased transcription, while the red down arrow symbol represents decreased transcription.

The results did not show significantly different levels of the relative expression of the investigated markers between the different compartments (the cortex vs. medulla). The eventual explanation for this could be high energy demand of both tissues. Moreover, the mitochondria in the cells of both parts of the adrenal gland (the cortex and medulla) are important for the synthesis of stress hormones (glucocorticoids and catecholamines) since some steps of biosynthesis take place in mitochondria. The present results revealed that most markers related to mitochondrial dynamics exhibited altered transcriptional profiles and clearly showed a distinction in the transcriptional profiles between some of the members of the same family (*Ppara* vs. *Ppard*) in the adrenal glands obtained from acutely (1 × IMO) and repeatedly (2 × IMO and 10 × IMO) stressed rats (Figure 9).

Specifically, in the whole adrenal gland, 81% (17/21) of the markers were altered, while in the adrenal cortex and adrenal medulla, the figures were 76.2% (16/21) and 85.7% (18/21), respectively. The changes were mostly up-regulation (please see the green arrows in Figure 9) and were observed in the transcriptional profile of the markers of every process of mitochondrial dynamics, including the markers of mitobiogenesis, mitofusion, and mitophagy. It is clearly evident that the profiles of these changes were similar in the whole adrenal gland, the cortex, and the medulla. The transcription of the genes (*Ppargc1a*, *Ppargc1b*) encoding the protein master regulator of mitochondrial dynamics and integrator of environmental signals PGC1 [1,2,3,4] significantly decreased in the adrenal glands of the stressed rats. In the whole adrenal gland, 62.5% (5/8) of the mitobiogenesis markers were altered, while in the adrenal cortex and adrenal medulla, the figures were 62.5% (5/8) and 87.5% (7/8), respectively. The mitofusion markers were changed up to 100% (3/3) in the whole adrenal gland, 66.7% (5/8) in the adrenal cortex, and 87.5% (7/8) in the adrenal medulla. Additionally, most markers related to mitochondrial functionality were also altered, with figures of 83.3% (5/6), 83.3% (5/6), and 66.7% (4/6) in the whole adrenal gland, adrenal cortex, and adrenal medulla, respectively. Overall, these findings suggest that acute and repeated stress have a significant impact on mitochondrial dynamics in the adrenal gland. It is clearly evident that the profiles of these changes were similar in the whole adrenal gland, the cortex, and the medulla. The transcription of the genes (*Ppargc1a*, *Ppargc1b*) encoding the protein master regulator of mitochondrial dynamics and integrator of environmental signals PGC1 [1,2,3,4] significantly decreased in the adrenal glands of the stressed rats. However, the levels of one set of PGC1 targets (*Nrf1*, *Nrf2*, *Tfam*) remained unchanged. Interestingly, the other targets of PGC1 and the members of the same family (*Ppara* vs. *Ppard*) were oppositely affected by stress in the same samples: a significant reduction in *Ppara* was observed, while the expression of *Ppard* increased in response to all types of IMO. It is very difficult to explain these opposing effects since PPARa and PPARd are both nuclear receptors and transcription coactivators involved in the regulation of mitochondrial biogenesis, metabolism, inflammation, and energy homeostasis [23]. However, while both PPARa and PPARd are involved in the regulation of metabolism, inflammation, and energy homeostasis, they have different tissue distributions and physiological functions. Studies have suggested that PPARa may play a role in regulating the balance between glucocorticoid and mineralocorticoid production in the adrenal gland [24], while PPARd is more involved in inflammatory response [25]. Significant increases in *Ppard* correlated with the levels of the stress hormones corticosterone (0.969) and adrenaline (0.981). On the other hand, the decrease in the *Ppara* transcript could be a consequence of the reduction in the level of AMPK protein in the adrenal glands of repeatedly stressed rats. The transcription of all markers of mitochondrial fusion (*Mfn1*, *Mfn2*, *Opa1*) and fission (*Drp1*, *Fis1*) increased with the treatment with ten times repeated IMO. The increase in *Mfn2* correlated with the levels of stress hormones corticosterone (0.882) and adrenaline (0.878). These changes could contribute to alterations in mitochondrial morphology and network organization, as well as steroid hormones synthesis. Namely, it has been shown that MFN2 expression is required for StAR, ERK, and MEK mitochondrial localization and for aldosterone synthesis in human adrenocortical cells. On the other hand, it was suggested that angiotensin-II is involved in the regulation of *Mfn2* in human adrenocortical cells [26]. The treatment with ten repeated incidents of IMO oppositely affected the transcription of the genes (*Pink1*, *Prkn*) encoding the proteins involved in the regulation of mitophagy, a process that clears damaged or dysfunctional mitochondria, enabling proper mitochondrial quality control. The *Pink1* transcripts increased, while the *Prkn* transcripts decreased. The increase in *Pink1* correlated with the levels of the stress hormones corticosterone (0.879) and adrenaline (0.881). Moreover, the expression of transcripts related to mitochondrial function, such as *Cytc*, *Cox4i1,* and *Cox4i2*, significantly increased in the adrenal glands of rats exposed to ten times repeated IMO, indicating an attempt to compensate for decreased mitochondrial function. The significant increase in *Cytc* correlated with the levels of the stress hormones corticosterone (0.941) and adrenaline (0.970). However, the expression of the transcript for the gene (*Ucp1*) encoding the protein involved in uncoupling oxidative phosphorylation significantly decreased in the adrenal glands of rats exposed to acute and two repeated incidents of IMO, while *Ucp1* transcripts increased dramatically in the adrenal glands of rats exposed to ten times repeated IMO, suggesting that acute stress and two times repeated stress may activate some adaptive mechanisms related to thermogenic mitochondrial metabolism. In the same samples, the levels of the *Ucp3* transcripts were significantly increased by all types of IMO, suggesting a complex regulation of energy metabolism in the adrenal gland, which is known to have an extremely high energy demand. UCP1 and UCP3 are two members of the uncoupling protein family that are involved in the regulation of energy metabolism and thermogenesis in different tissues. While both UCP1 and UCP3 are involved in the regulation of energy metabolism and mitochondrial function, they have distinct tissue distributions and physiological functions. UCP1 is primarily involved in the regulation of thermogenesis in brown adipose tissue, while UCP3 is expressed in a variety of tissues and its functions are not yet fully understood. Recently, it was shown that the mouse adrenal gland is a novel organ expressing UCP1, and its expression is not upregulated by cold exposure [27].

The limitation of this study is that the mechanism(s) involved in the shaping of the transcriptional profiles of the markers of mitochondrial dynamics in the adrenal glands of adult male rats are not shown. However, our preliminary result using an agonist and/or antagonist of stress hormones and their receptors showed that some of the effects are direct consequences of the activation of specific types of the adrenergic receptor (unpublished results). Accordingly, further studies are needed to elucidate the underlying mechanisms and potential implications of disturbed mitochondrial dynamics on health and disease.

## 5. Conclusions

This study revealed that acute and repeated stress significantly change the transcriptional profiles of mitochondrial dynamics in the adrenal glands of adult male rats, and there was a clear distinction in the transcriptional profiles between some of the members of the same family (*Ppara* vs. *Ppard*) in the adrenal glands of the stressed rats.

## Data Availability

All relevant data and samples are available from the corresponding author on request. Further information and requests for resources and reagents should be directed to and will be fulfilled by the lead contact, Silvana Andric (silvana.andric@dbe.uns.ac.rs).

## Supplementary Materials

The following supporting information can be downloaded at: https://www.mdpi.com/article/10.3390/life13071457/s1, Supplementary Material And Methods: Table S1: Key resources table; Table S2: Primers sequences used for the real-time PCR analysis of molecular markers of mitochondrial biogenesis; Table S3: Primers sequences used for the real-time PCR analysis of molecular markers of mitochondrial fusion and architecture; Table S4: Primers sequences used for the real-time PCR analysis of molecular markers of mitochondrial fission; Table S5: Primers sequences used for the real-time PCR analysis of molecular markers of mitochondrial autophagy; Table S6: Primers sequences used for the real-time PCR analysis of molecular markers of mitochondrial functionality; Table S7. Primers sequences used for the real-time PCR analysis of adrenergic receptor, adrenergic receptor kinases and glucocorticoid receptor; Table S8. The characteristics of the antibodies; Histological analyses. Supplementary Results: Figure S1: Weight of the whole adrenal glands of stressed adult male rats; Figure S2: Fascicular layer of adrenals of control (A) and stressed animal (B); Table S9: The levels of stress hormones in circulation/serum; Figure S3: ΔCt values of the main mitochondrial biogenesis markers in the whole adrenal gland, adrenal cortex, and medulla tissues after one-time, two-times and ten-times repeated psychophysical stress of the entire organism; Figure S4: ΔCt values of the main mitochondrial fusion and architecture markers in the whole adrenal gland, adrenal cortex and medulla tissues after one-time, two-times and ten-times repeated psychophysical stress of the entire organism; Figure S5: ΔCt values of the main mitochondrial fission markers in the whole adrenal gland, adrenal cortex and medulla tissues after one-time, two-times and ten-times repeated psychophysical stress of the entire organism; Figure S6: ΔCt values of the main mitochondrial autophagy markers in the whole adrenal gland, adrenal cortex and medulla tissues after one-time, two-times and ten-times repeated psychophysical stress of the entire organism; Figure S7: ΔCt values of the main mitochondrial functionality markers in the whole adrenal gland, adrenal cortex and medulla tissues after one-time, two-times and ten-times repeated psychophysical stress of the entire organism; Supplementary Figure S8: ΔCt values of the adrenergic receptors, adrenergic receptor kinases and glucocorticoid receptor in the whole adrenal gland, adrenal cortex and medulla tissues after one-time, two-times and ten-times repeated psychophysical stress of the entire organism.
