# Supplementary material for: Transcriptional Profiles of Mitochondrial Dynamics Markers Are Disturbed in Adrenal Glands of Stressed Adult Male Rats
Source: Life (Basel). 2023 Jun 27;13(7):1457. doi: 10.3390/life13071457 (PMC10381216; doi:10.3390/life13071457)
Supplement: Supplementary file 1 [file life-13-01457-s001.zip › life-2403503-supplementary.pdf]

SUPPLEMENTARY

Manuscript Title

Transcriptional Profiles of Mitochondrial Dynamics Markers Are Disturbed in Adrenal Glands of Stressed Adult Male Rats

Authors

Isidora M. Keselj [1], Filip N. Bozic [1], Miodrag M. Vucinic [1], Dusan Lalosevic [2], Tatjana S. Kostic [1] and Silvana A. Andric [1,*]

Affiliation

[1]Laboratory for Reproductive Endocrinology and Signaling, Laboratory for Chronobiology and Aging, CeRES, DBE, Faculty of Sciences, University of Novi Sad, 21000 Novi Sad, Serbia

[2]Medical Faculty, University of Novi Sad, 21000 Novi Sad, Serbia

***Correspondence**

Silvana A Andric, PhD, professor
Head of Laboratory for Reproductive Endocrinology and Signaling
Member of Laboratory for Chronobiology and Aging
Head of accredited Center of Excellence for Reproductive Endocrinology
Faculty of Sciences, University of Novi Sad
Dositeja Obradovica Square 2, 21000 Novi Sad, Serbia
Tel. +381-21-485-2673 (office); +381-63-748-4403 (mobile); Fax: +381-21-450-620
E-mail: silvana.andric@dbe.uns.ac.rs
Web: *http://wwwold.dbe.pmf.uns.ac.rs/en/nauka-eng/lares*

Supplementary information file contains two sections, supplementary material and methods section and supplementary results section.

SUPPLEMENTARY MATERIAL AND METHODS

Supplementary Table S1. Key resources table

Resource or reagent	Source	Identifier
**Experimental model and biological samples**		
Wistar rat	LaRES and ChronAge Laboratories (DBE, Faculty of Sciences, University of Novi Sad, Serbia)	*http://wwwold.dbe.pmf.uns.ac.rs/en/nauka-eng/lares*
Adrenal gland	Three months-old male rats	NA
**Commercial Reagents/Assays**		
TRIzol™ Reagent	Invitrogen™, Thermo Fisher Scientific, USA	*https://www.thermofisher.com*
DNase I (RNase-free) treatment	New England Biolabs, USA	*https://international.neb.com*
High Capacity Kit for cDNA	Applied Biosystems/Thermo Fisher Scientific, USA	*https://www.thermofisher.com*
Power SYBR® Green PCR Master Mix	Applied Biosystems/Thermo Fisher Scientific, USA	*https://www.thermofisher.com*
**Primers**		
Supplementary Tables S2 to S7	This paper	*www.ncbi.nlm.nih.gov/sites/entrez*
**Characteristics of the antibodies**		
Supplementary Table S8	This paper	
**Software**		
GraphPad Prism 8 Software	GraphPad Prism	*https://www.graphpad.com/scientific-software/prism*

Supplementary Table S2. Primers sequences used for the real-time PCR analysis of molecular markers of mitochondrial biogenesis.

Gene	Accession code	Primers	Primer length	Product length	AV Ct		
					Whole	Cortex	Medulla
Ppargc1a	NM_031347	F: 5'-AGCCGTAGGCCCAGGTATGACA-3'<br>R: 5'-TGCTTGGCCCTTTCAGACTCCC-3'	22 bp<br>22 bp	107 bp	28.37	28.80	27.60
Ppargc1b	NM_176075	F: 5'-ACCTTCCGGTGTTCGGAGCATG-3'<br>R: 5'-GTGGAAGGAGGGCTCATTGCGT-3'	22 bp<br>22 bp	81 bp	29.12	28.10	27.95
Tfam	NM_031326	F: 5'-TATAGTCGTCGGCCCGAGGGAT-3'<br>R: 5'-AAGGCTGACAGGCGAGGGTATG-3'	22 bp<br>22 bp	125 bp	26.31	25.65	26.37
Nrf1	NM_0011007<br>08	F: 5'-GACCATCCAGACGACGCAAGCA-3'<br>R: 5'-ATGGGCGGCAGCTTCACTGTT-3'	22 bp<br>21 bp	136 bp	26.19	26.44	26.01
Nrf2a	NM_0011088<br>41	F: 5'-AGCGGAACTGAACCGCTTGGT-3'<br>R: 5'-GTGACTGGCTGAGCAATCCCGT-3'	21 bp<br>22 bp	84 bp	24.55	24.34	24.14
Ppara	NM_013196	F: 5'-GTCCTGGAACTGAAGCGACGCT-3'<br>R: 5'-TTACGCCCAAATGCACCACGC-3'	22 bp<br>21 bp	110 bp	29.37	29.16	28.48
Ppard	NM_013141	F: 5'-ACGGTAAAGGCGGTCCATCTGC-3'<br>R: 5'-TCCTCCTGTGGCTGTTCCATGAC-3'	22 bp<br>23 bp	109 bp	26.78	26.82	25.00
mtNd1		F: 5' GCGTGGGAGGAGCATCAGGG 3'<br>R: 5' GCGAATGGTCCTGCGGCGT A 3'	20 bp<br>20 bp	271 bp	17.46	17.22	17.88
Gapdh	NM_017008	F: 5'-TGCCAAGTATGATGACATCAAGAAG-3'<br>R: 5'-AGCCCAGGATGCCCTTTAGT-3'	25 bp<br>20 bp	110 bp	18.89	19.00	20.48

Primers were designed by using software Primer Express 3.0 (Applied Biosystems) and full genes sequences from the NCBI Entrez Nucleotide database (*www.ncbi.nlm.nih.gov/sites/entrez*). F - forward; R - reverse.

Supplementary Table S3. Primers sequences used for the real-time PCR analysis of molecular markers of mitochondrial fusion and architecture.

Gene	Accession code	Primers	Primer length	Product length	AV Ct		
					Whole	Cortex	Medulla
Mfn1	NM_138976.1	F: 5'-CCTTGTACATCGATTCCTGGGTTC-3'<br>R: 5'-CCTGGGCTGCATTATCTGGTG-3'	24 bp<br>21 bp	143 bp	21.68	22.71	23.37
Mfn2	NM_130894.4	F: 5'-TCAAGCGCCAGTTTGTGGAG-3'<br>R: 5'-CACAGATGAGCAAATGTCCCAGA-3'	20 bp<br>23 bp	118 bp	22.02	22.78	23.20
Opa1	NM_133585.3	F: 5'-AAAAGCCCTTCCCAGTTCAGA-3'<br>R: 5'-TACCCGCAGTGAAGAAATCCTT-3'	21 bp<br>22 bp	101 bp	21.79	22.17	22.84
Gapdh	NM_017008	F: 5'-TGCCAAGTATGATGACATCAAGAAG-3'<br>R: 5'-AGCCCAGGATGCCCTTTAGT-3'	25 bp<br>20 bp	110 bp	16.59	17.45	17.45

Primers were designed by using software Primer Express 3.0 (Applied Biosystems) and full genes sequences from NCBI Entrez Nucleotide database (*www.ncbi.nlm.nih.gov/sites/entrez*). F - forward; R - reverse.

Supplementary Table S4. Primers sequences used for the real-time PCR analysis of molecular markers of mitochondrial fission.

Gene	Accession code	Primers	Primer length	Product length	AV Ct		
					Whole	Cortex	Medulla
Fis1	NM_001105919.1	F: 5'-ACGCCTGCCGTTACTTCTTC-3'<br>R: 5'-GCAACCCTGCAATCCTTCAC-3'	20 bp<br>20 bp	108 bp	22.79	24.62	24.60
Drp1	NM_053655.3	F: 5'-AGGTTGCCCGTGACAAATGA-3'<br>R: 5'-CACAGGCATCAGCAAAGTCG-3'	20 bp<br>20 bp	94 bp	22.02	22.73	23.55
Gapdh	NM_017008	5'-TGCCAAGTATGATGACATCAAGAAG-3'<br>R: 5'-AGCCCAGGATGCCCTTTAGT-3'	25 bp<br>20 bp	110 bp	16.59	17.45	17.45

Primers were designed by using software Primer Express 3.0 (Applied Biosystems) and full genes sequences from NCBI Entrez Nucleotide database (*www.ncbi.nlm.nih.gov/sites/entrez*). F - forward; R - reverse.

Supplementary Table S5. Primers sequences used for the real-time PCR analysis of molecular markers of mitochondrial autophagy.

Gene	Accession code	Primers	Primer length	Product length	AV Ct		
					Whole	Cortex	Medulla
Pink1	NM_0011066 94.1	F: 5'-CAAGCAAGTGTCTGACCCAC-3'<br>R: 5'-GCTTCATACACAGCGGCATT-3'	20 bp<br>20 bp	111 bp	21.12	21.16	21.98
Prkn	NM_020093. 1	F: 5'-CTTCCAGCTCAAGGAAGTGG-3'<br>R: 5'-CAGAGGCATTTGTTTCGTGA-3'	20 bp<br>20 bp	182 bp	24.74	28.94	26.34
Gapdh	NM_017008	F: 5'-TGCCAAGTATGATGACATCAAGAAG-3'<br>R: 5'-AGCCCAGGATGCCCTTTAGT-3'	25 bp<br>20 bp	110 bp	16.59	17.45	17.45

Primers were designed by using software Primer Express 3.0 (Applied Biosystems) and full genes sequences from NCBI Entrez Nucleotide database (*www.ncbi.nlm.nih.gov/sites/entrez*). F - forward; R - reverse.

Supplementary Table S6. Primers sequences used for the real-time PCR analysis of molecular markers of mitochondrial functionality.

Gene	Accession code	Primers	Primer length	Product length	AV Ct		
					Whole	Cortex	Medulla
Cox4i1	NM_017202	F: 5'-CGCTGAGATGAACAAGGGCACC-3'<br>R: 5'-TCCCAGATCAGCACAAGCGCA-3'	22 bp<br>21 bp	93 bp	21.06	22.45	20.48
Cox4i2	NM_053472	F: 5'-CACAGCCCAGGAAGTGCTGCTA-3'<br>R: 5'-TGTGCAGTAAGGCTCATCCGGC-3'	22 bp<br>22 bp	105 bp	27.33	29.11	27.02
Cytc	NM_012839	F:5'-GCAAGCATAAGACTGGACCAAA-3'<br>R:5'-TTGTTGGCATCTGTGTAAGAGAATC-3'	22 bp<br>25 bp	88 bp	21.87	23.22	22.39
Ucp1	NM_012682	F: 5'-TCAGCTCTTGTCGCCGGGTTT-3'<br>R: 5'-TGCACAGCTGGGTACACTTGGG-3'	21 bp<br>22 bp	114 bp	30.77	28.54	32.52
Ucp2	NM_019354	F: 5'-ACGACCTCCCTTGCCACTTCAC-3'<br>R: 5'-GGTACTGGCCCAAGGCAGAGTT-3'	22 bp<br>22 bp	117 bp	19.60	19.59	20.32
Ucp3	NM_013167	F: 5'-TGCTCAACCCACGGATGTGGT-3'<br>R: 5'-CCTGGCGATGGTTCTGTAGGCA-3'	21 bp<br>22 bp	112 bp	26.54	28.05	27.44
Gapdh	NM_017008	F: 5'-TGCCAAGTATGATGACATCAAGAAG-3'<br>R: 5'-AGCCCAGGATGCCCTTTAGT-3'	25 bp<br>20 bp	110 bp	18.89	19.00	20.48

Primers were designed by using software Primer Express 3.0 (Applied Biosystems) and full genes sequences from the NCBI Entrez Nucleotide database (*www.ncbi.nlm.nih.gov/sites/entrez*). F - forward; R – reverse.

Supplementary Table S7. Primers sequences used for the real-time PCR analysis of adrenergic receptor, adrenergic receptor kinases and glucocorticoid receptor.

Gene	Accession code	Primers	Primer length	Product length	AV Ct		
					Whole	Cortex	Medulla
Adra1d	NM_024483	F: 5′-GAAGGTGATGGGTTATGGTG-3′<br>R: 5′-GAAGCCATAGCTGAAGCCT-3′	20 bp<br>19 bp	152 bp	27.27	26.65	26.52
Adrb1	NM_012701	F: 5′-TGCCCATCCTCATGCACTGGTG-3′<br>R: 5′-GTAGGCCCGGTTGGTGACGAAA-3′	22 bp<br>22 bp	98 bp	32.81	30.46	26.98
Adrb2	NM_012492	F: 5′-CGCCCTTCAAGTACCAGAGCCT-3′<br>R: 5′-TTGCTTGTGGGTGGCACGGT-3′	22 bp<br>20 bp	131 bp	28.55	28.20	26.19
Adrbk1	NM_012776	F: 5′-GCAGCGAGTGCCCAAGATGAAG-3′<br>R: 5′-CACTGCCACGCTGAATCAGTGG-3′	22 bp<br>22 bp	83 bp	25.29	25.75	28.72
Adrbk2	NM_012897	F: 5′-GAGCTGACGTGCACCTTCAACG-3′<br>R: 5′-GATGGCGGCACGTGGTTTGT-3′	22 bp<br>20 bp	81 bp	27.42	28.52	27.87
Nr3c1	NC_005117.4	F:5'-CGGTTAATCTGCACAGCCTAT-3'<br>R:5'-AAAATGGGTCGGTGCTTCTA-3'	21 bp<br>20 bp	147 bp	24.91	26.23	24.57
Gapdh	NM_017008	F: 5'-TGCCAAGTATGATGACATCAAGAAG-3'<br>R: 5'-AGCCCAGGATGCCCTTTAGT-3'	25 bp<br>20 bp	110 bp	18.86	19.16	19.08

Primers were designed by using software Primer Express 3.0 (Applied Biosystems) and full genes sequences from the NCBI Entrez Nucleotide database (*www.ncbi.nlm.nih.gov/sites/entrez*). F - forward; R – reverse.

Supplementary Table S8. The characteristics of the antibodies.

Target	Name of Antibody	Antigen sequence	Manufacturer, catalog #	Mono- or polyclonal	Dil. used
PGC1 alpha	PGC1 alpha polyclonal antibody	Synthetic peptide CVRTYWLLGERGCSTRG (C terminal)	Invitrogen Catalog # PA1-31202 Mw (PGC-1) = 91 kDa	Rabbit polyclonal antibody	1:500
MFN1	Mfn1 (D-10): sc-166644	Antibody raised against amino acids 10-74 mapping within an N-terminal cytoplasmic domain of Mfn1 of human origin.	Santa Cruz Biotechnology Inc. sc-166644 Mw (MFN1) = 86 kDa	Mouse monoclonal antibody	1:100
MFN2	Mfn2 (F-5): sc-515647	Antibody raised against amino acids 461-528 mapping within a cytoplasmic domain of Mfn2 of human origin	Santa Cruz Biotechnology Inc. sc-515647 Mw (MFN2) = 86 kDa	Mouse monoclonal antibody	1:100
PRKAc	Purified Mouse Anti-PKA[C]	Humaofjfjn PKA[Cα] subunit aa. 18-347	BD Transduction Laboratories™ 610980 MW (PRKAc)= 40 kDa	Mouse monoclonal antibody	1:500
AMPKα1/2	AMPK alpha 1/2 Antibody (D-6): sc-74461	Antibody raised against amino acids 251-550 mapping at the C-terminus of AMPKα1 of human origin	Santa Cruz Biotechnology Inc. sc-74461 Mw (AMPK alpha 1/2) = 63 kDa	Mouse monoclonal antibody	1:100
ACTIN	Actin (C-2): sc-8432	Antibody specific for an epitope mapping between amino acids 350-375 at the C-terminus of Actin of human origin	Santa Cruz Biotechnology Inc. sc-8432 Mw (ACTIN) = 43 kDa	Rabbit polyclonal antibody	1:100

Histological analysis

Isolated adrenal glands were fixed in Bouin's solution; the 5 mm thick sections were dehydrated in isopropyl alcohol, embedded in paraffin, and cut on a rotary microtome (Leica, Germany) at 5 μm. Hematoxylin-eosin (H&E) staining was performed, and visualization was done by Leica optical microscope with digital camera.

SUPPLEMENTARY RESULTS

Repeated stress increased weight of the whole adrenal glands.

After psychophysical strees by immobilization of adult male rats, whole adrenal glands were isolated and measured. Results show increased weight of adrenal gland, where statisticaly significant increase was detected in 2xIMO and 10xIMO groups (Supplementary Figure S1).

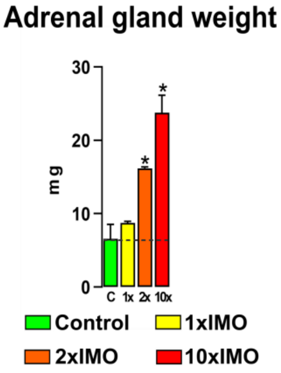

Supplementary Figure S1. Weight of the whole adrenal glands of stressed adult male rats.

Adult male rats were subjected to psychophysical stress by immobilization. Briefly, stressed (IMO) rats were bound in a supine position to a wooden board by fixing the rats' limbs using thread, while the head motion was not limited. In each experiment, unstressed, freely moving animals were used as a control group. To analyze the effect of psychophysical stress, animals were subjected to immobilization stress once (1xIMO) for two hours, or repeatedly for two (2xIMO) and ten (10xIMO) consecutive days, in the duration of two hours at the same period during the day (from 07:00 AM to 9:00 AM). At the end of the experimental period, control and stressed animals were quickly decapitated without anesthesia, and adrenal glands were isolated. After isolation, adrenal gland were measured.

Fascicular layer of adrenals of control and stressed animals.

Histological structure of stressed and control animals is generaly preserved, but in stressed group a reduction of zona glomerulosa and reduction of lipid droplets in zona fasciculata are noted. Those changes represent activation of adrenals under stress, especially in fascicular layer.

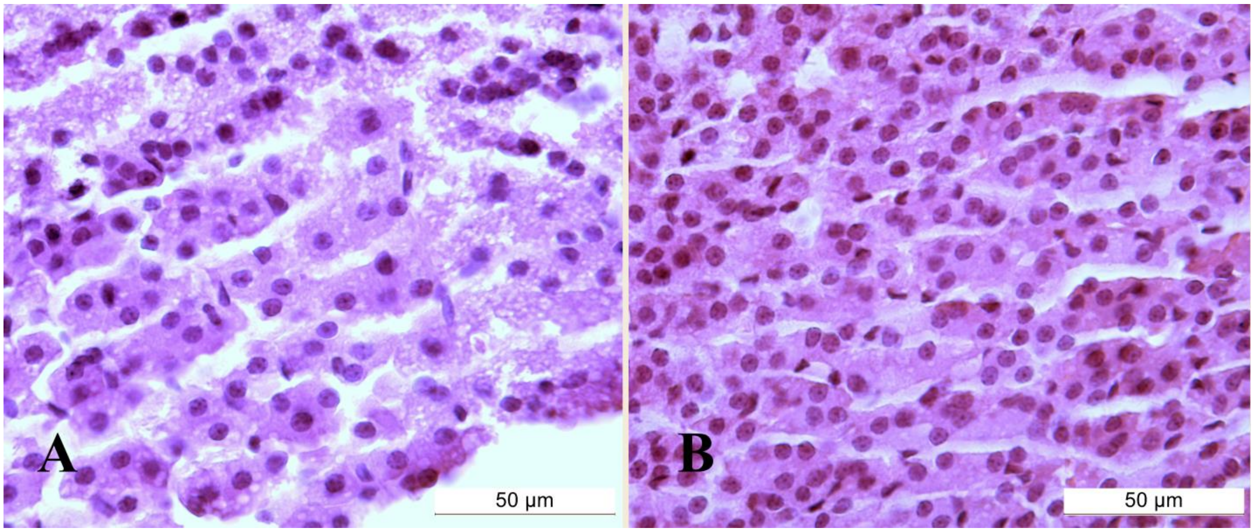

Supplementary Figure S2. Fascicular layer of adrenals of control (A) and stressed animal (B).

To analyze the effect of psychophysical stress, adult male rats were subjected to immobilization stress once (1xIMO) for two hours, or repeatedly for two (2xIMO) and ten (10xIMO) consecutive days, in the duration of two hours at the same period during the day (from 07:00 AM to 9:00 AM). In each experiment, unstressed, freely moving animals were used as a control group. At the end of the experimental period, control and stressed animals were quickly decapitated without anesthesia, and adrenal glands were isolated. Isolated adrenal glands were fixed in Bouin's solution; the 5 mm thick section was dehydrated in isopropyl alcohol, embedded in paraffin, and cut on a rotary microtome (Leica, Germany) at 5 μm. Hematoxylin-eosin (H&E) staining was performed, and visualization was done by Leica optical microscope with digital camera.

Immobilization stress disturbs circulating stress hormones

To mimic psychophysical stress, adult male rats were subjected to immobilization stress once (1xIMO) for two hours, or repeatedly for two (2xIMO) and ten (10xIMO) consecutive days. In each experiment, unstressed, freely moving animals were used as a control group. At the end of the experimental period, control and stressed animals were quickly decapitated without anesthesia and trunk blood was collected. To verify the functionality of the treatment used in the present study, concentration of stress hormones corticosterone and adrenaline were measured in serum collected from undisturbed (control) and stressed animals. Results show that IMO was effective as a stressor (Supp. Figure 1), elevating serum adrenaline and CORT levels in all stressed groups comparing to undisturbed controls.

Group	Adrenaline [pg/ml]		CORT [pmol/ml]	
	AV	SEM	AV	SEM
**Control**	51.01	2.02	12.87	0.95
**1xIMO**	156.43	5.71	58.80	2.04
**2xIMO**	132.21	4.92	80.00	3.16
**10xIMO**	111.25	5.01	52.90	1.97

Supplementary Table S9. IMO disturbs circulating levels of stress hormones adrenaline and corticosterone.

ΔCt values of the main mitochondrial biogenesis markers.

Analysis of relative expression of main mitochondrial biogenesis markers was done using delta delta Ct (ΔΔCt) method. In order to analyze difference in relative expression levels of main mitochondrial biogenesis markers in the whole adrenal gland, adrenal cortex and adrenal medulla, ΔCt values were used. Results show that there is no statistical difference in ΔCt values of all the analyzed mitochondrial biogenesis markers (Supplementary Figure S3).

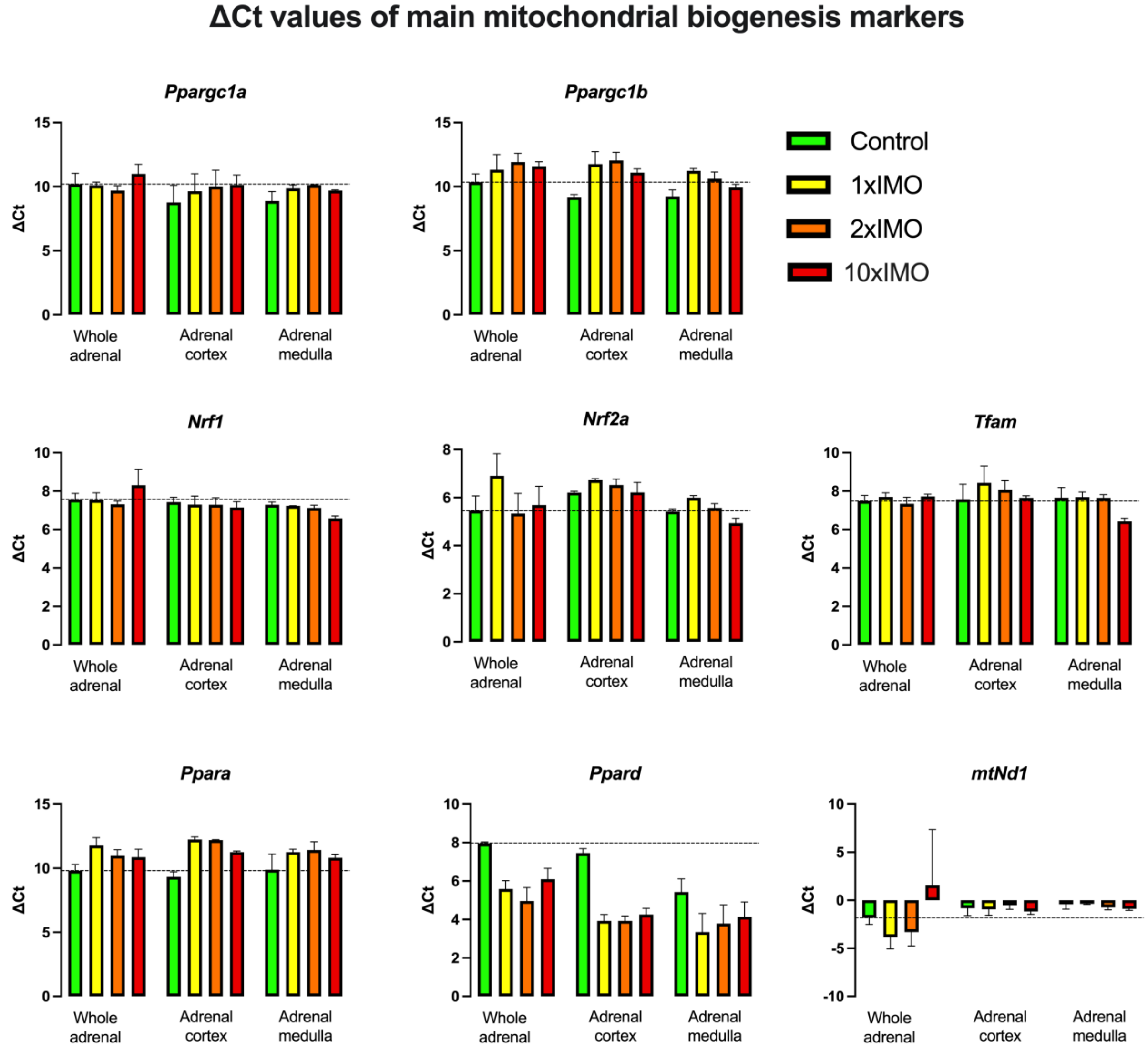

Supplementary Figure S3. ΔCt values of the main mitochondrial biogenesis markers in the whole adrenal gland, adrenal cortex and medulla tissues after one-time, two-times and ten-times repeated psychophysical stress of the entire organism.

Whole adrenal gland tissue, as well as the adrenal cortex and medulla tissues, were isolated from undisturbed, acutely (1xIMO) and repeatedly (2xIMO and 10xIMO) stressed rats. Tissue was further used for RNA isolation followed by the analysis of relative expression of mitochondrial biogenesis markers. The data bars represent mean ± SEM values of two independent *in vivo* experiments.

ΔCt values of the main mitochondrial fusion and architecture markers.

Analysis of relative expression of main mitochondrial fusion and architecture markers was done using delta delta Ct (ΔΔCt) method. In order to analyze difference in relative expression levels of main fusion and architecture markers in the whole adrenal gland, adrenal cortex and adrenal medulla, ΔCt values were used. Results show that there is no statistical difference in ΔCt values of all the analyzed fusion and architecture (Supplementary Figure S4).

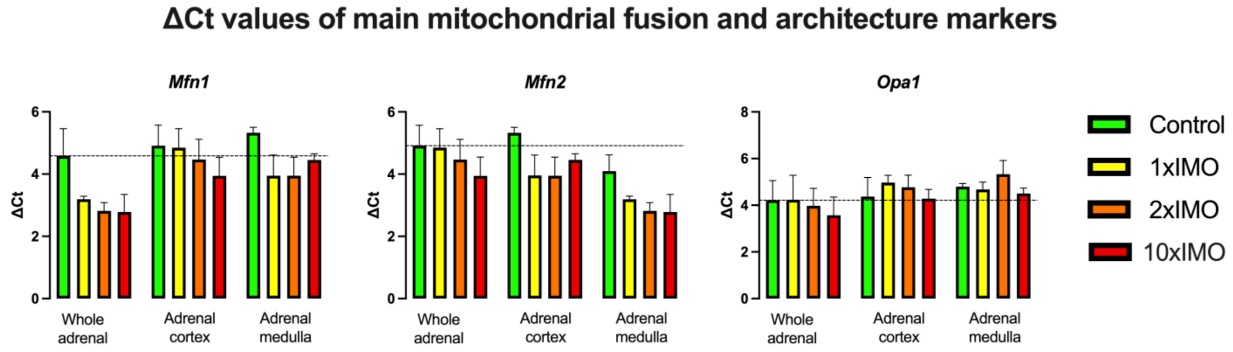

Supplementary Figure S4. ΔCt values of the main mitochondrial fusion and architecture markers in the whole adrenal gland, adrenal cortex and medulla tissues after one-time, two-times and ten-times repeated psychophysical stress of the entire organism.

Whole adrenal gland tissue, as well as the adrenal cortex and medulla tissues, were isolated from undisturbed, acutely (1xIMO) and repeatedly (2xIMO and 10xIMO) stressed rats. Tissue was further used for RNA isolation followed by the analysis of relative expression of mitochondrial fusion and architecture markers. The data bars represent mean ± SEM values of two independent *in vivo* experiments.

ΔCt values of the main mitochondrial fission markers.

Analysis of relative expression of main mitochondrial fission markers was done using delta delta Ct (ΔΔCt) method. In order to analyze difference in relative expression levels of main markers of mitochondrial fission in the whole adrenal gland, adrenal cortex and adrenal medulla, ΔCt values were used. Results show that there is no statistical difference in ΔCt values of all the analyzed mitochondrial fission markers (Supplementary Figure S5).

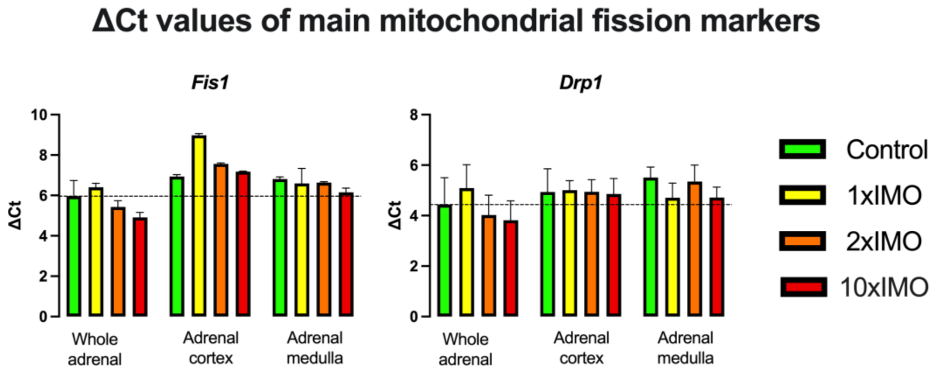

Supplementary Figure S5. ΔCt values of the main mitochondrial fission markers in the whole adrenal gland, adrenal cortex and medulla tissues after one-time, two-times and ten-times repeated psychophysical stress of the entire organism.

Whole adrenal gland tissue, as well as the adrenal cortex and medulla tissues, were isolated from undisturbed, acutely (1xIMO) and repeatedly (2xIMO and 10xIMO) stressed rats. Tissue was further used for RNA isolation followed by the analysis of relative expression of mitochondrial fission markers. The data bars represent mean ± SEM values of two independent *in vivo* experiments.

ΔCt values of the main markers of mitophagy.

Analysis of relative expression of main mitochondrial autophagy markers was done using delta delta Ct (ΔΔCt) method. In order to analyze difference in relative expression levels of main markers of mitochondrial autophagy in the whole adrenal gland, adrenal cortex and adrenal medulla, ΔCt values were used. Results show that there is no statistical difference in ΔCt values of all the analyzed mitochondrial autophagy markers (Supplementary Figure S6).

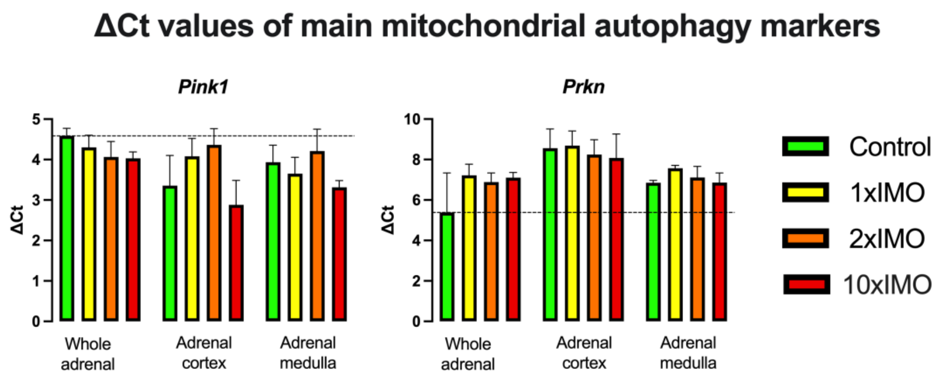

Supplementary Figure S6. ΔCt values of the main mitochondrial autophagy markers in the whole adrenal gland, adrenal cortex and medulla tissues after one-time, two-times and ten-times repeated psychophysical stress of the entire organism.

Whole adrenal gland tissue, as well as the adrenal cortex and medulla tissues, were isolated from undisturbed, acutely (1xIMO) and repeatedly (2xIMO and 10xIMO) stressed rats. Tissue was further used for RNA isolation followed by the analysis of relative expression of mitochondrial autophagy markers. The data bars represent mean ± SEM values of two independent *in vivo* experiments.

ΔCt values of the main mitochondrial functionality markers.

Analysis of relative expression of main mitochondrial functionality markers was done using delta delta Ct (ΔΔCt) method. In order to analyze difference in relative expression levels of main markers of mitochondrial functionality in the whole adrenal gland, adrenal cortex and adrenal medulla, ΔCt values were used. Results show that there is no statistical difference in ΔCt values of all the analyzed mitochondrial functionality markers (Supplementary Figure S7).

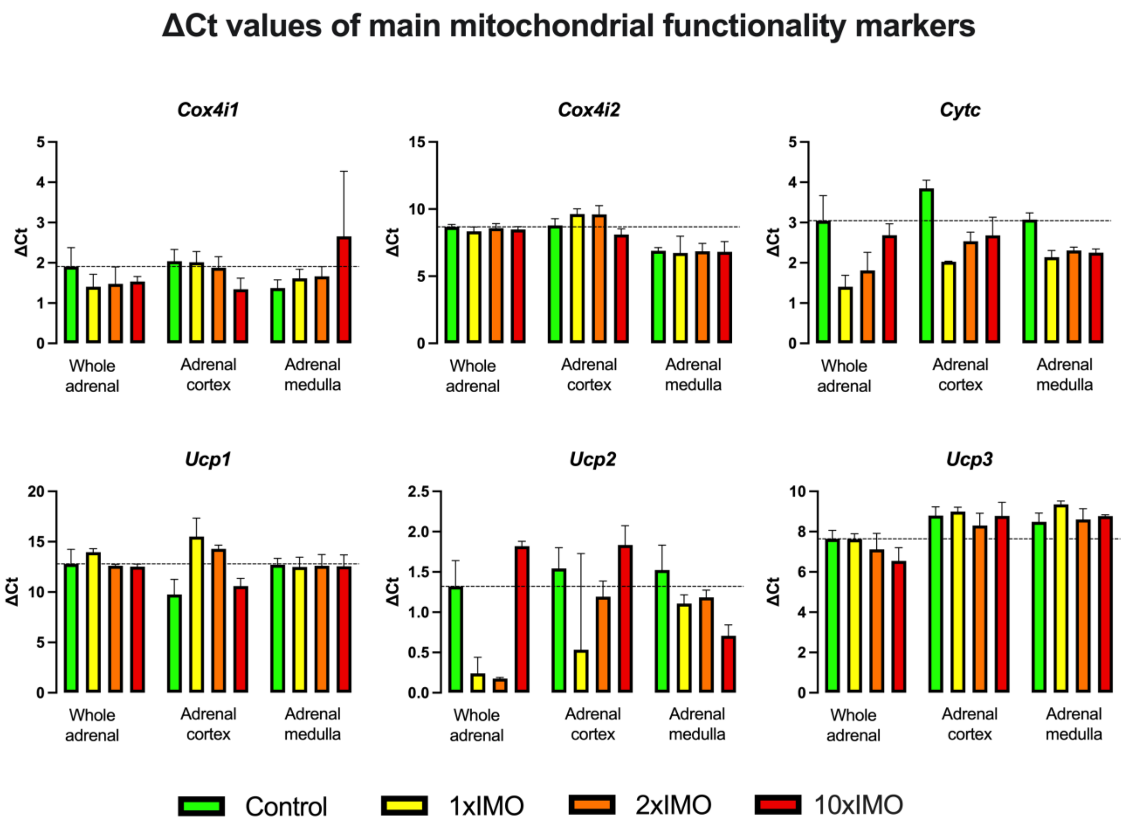

Supplementary Figure S7. ΔCt values of the main mitochondrial functionality markers in the whole adrenal gland, adrenal cortex and medulla tissues after one-time, two-times and ten-times repeated psychophysical stress of the entire organism.

Whole adrenal gland tissue, as well as the adrenal cortex and medulla tissues, were isolated from undisturbed, acutely (1xIMO) and repeatedly (2xIMO and 10xIMO) stressed rats. Tissue was further used for RNA isolation followed by the analysis of relative expression of mitochondrial functionality markers. The data bars represent mean ± SEM values of two independent *in vivo* experiments.

ΔCt values of adrenergic receptors, adrenergic receptor kinases and glucocorticoid receptor.

Analysis of relative expression of main mitochondrial functionality markers was done using delta delta Ct (ΔΔCt) method. In order to analyze difference in relative expression levels of main markers of adrenergic receptors and adrenergic receptor kinases (A) and glucocorticoid receptor (B) in the whole adrenal gland, adrenal cortex and adrenal medulla, ΔCt values were used. Results show that there is no statistical difference in ΔCt values of all the analyzed markers (Supplementary Figure S8).

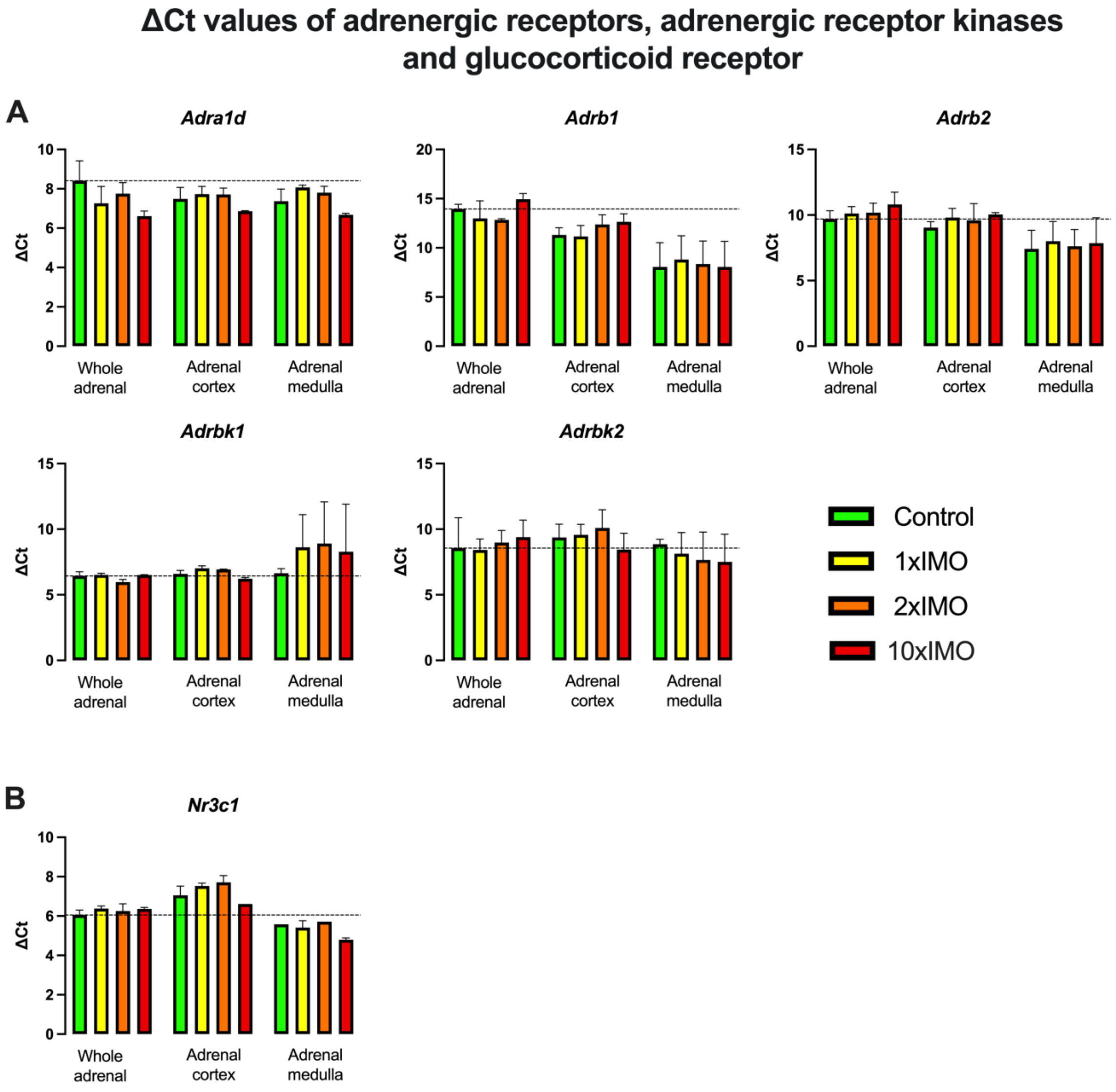

Supplementary Figure S8. ΔCt values of the adrenergic receptors, adrenergic receptor kinases and glucocorticoid receptor in the whole adrenal gland, adrenal cortex and medulla tissues after one-time, two-times and ten-times repeated psychophysical stress of the entire organism.

Whole adrenal gland tissue, as well as the adrenal cortex and medulla tissues, were isolated from undisturbed, acutely (1xIMO) and repeatedly (2xIMO and 10xIMO) stressed rats. Tissue was further used for RNA isolation followed by the analysis of relative expression adrenergic receptors and adrenergic receptor kinases (A) and glucocorticoid receptor (B). The data bars represent mean ± SEM values of two independent *in vivo* experiments.
